# N-Glycosylation of AXL Receptor Tyrosine Kinase Regulates Its Stability, Phosphorylation, and Oncogenic Function

**DOI:** 10.1016/j.mcpro.2026.101574

**Published:** 2026-04-27

**Authors:** Li Wang, Lingrui Li, Jidong Wang, Kishore Garapati, Feifei Li, Md Kamrul Hasan Khan, Xiangyi Kong, Hamid Bakshi, Dan Jiang, Shiya Zheng, Lifeng Chen, Jasper Yang, Xiaonan Hou, Scott H. Kaufmann, S. John Weroha, Jing Wang, Akhilesh Pandey, Xinyan Wu

**Affiliations:** 1 Department of Molecular Pharmacology and Experimental Therapeutics, Mayo Clinic, Rochester, Minnesota, USA; 2 Department of Breast Surgical Oncology, National Cancer Center/National Clinical Research Center for Cancer/Cancer Hospital, Chinese Academy of Medical Sciences and Peking Union Medical College, Beijing, China; 3 Department of gynecological oncology, Shandong Province Maternal and Child Healthcare Hospital, Jinan, Shandong, China; 4 Department of Laboratory Medicine and Pathology, Mayo Clinic, Rochester, Minnesota, USA; 5 Department of Gynecology, Nanjing Drum Tower Hospital, The Affiliated Hospital of Nanjing University Medical School, Nanjing University Medical School, Nanjing, China; 6 Department of Chemistry, Carleton College, Northfield, Minnesota, USA; 7 Department of Oncology, Mayo Clinic, Rochester, Minnesota, USA; 8 Center for Individualized Medicine, Mayo Clinic, Rochester, Minnesota, USA; 9 Manipal Academy of Higher Education (MAHE), Manipal, Karnataka, India

**Keywords:** N-glycosylation, AXL, receptor tyrosine kinase, proliferation, phosphorylation, proteomics

## Abstract

AXL, a receptor tyrosine kinase implicated in tumor progression, undergoes post-translational modifications that regulate its activity and stability. In this study, we demonstrated that AXL is extensively N-glycosylated in breast and ovarian cancer cells, existing predominantly as two isoforms corresponding to high-mannose and complex-type glycans. The extracellular cleaved soluble form of AXL (sAXL) primarily carries complex N-glycans and relies on glycosylation maturation. Functional analyses showed that complex glycosylation is essential for AXL membrane translocation and phosphorylation, as inhibition of N-glycan maturation caused cytoplasmic accumulation and impaired receptor activation. Mass spectrometry–based glycoproteomic analysis identified five glycosylation sites (N43, N157, N198, N339, and N345) with extensive microheterogeneity, including sialylation, fucosylation, and bisecting GlcNAc modifications. Evolutionary conservation analysis and mutational studies revealed that glycosylation at N43 and N339 is critical for AXL phosphorylation and cell proliferation, while multiple sites collectively contribute to protein stability and subcellular trafficking. Overall, our results demonstrate that N-glycosylation is essential for AXL stability, localization, and oncogenic signaling, offering new insights into the role glycosylation in regulating receptor tyrosine kinases function in cancer.

## Highlights

• AXL receptor tyrosine kinase carries both high-mannose and complex N-glycans
• N-glycosylation regulates AXL stability, localization, and oncogenic signaling.
• Glycosylation at N43 and N339 is critical for AXL phosphorylation and function.

## Graphical Abstract

**Figure:**
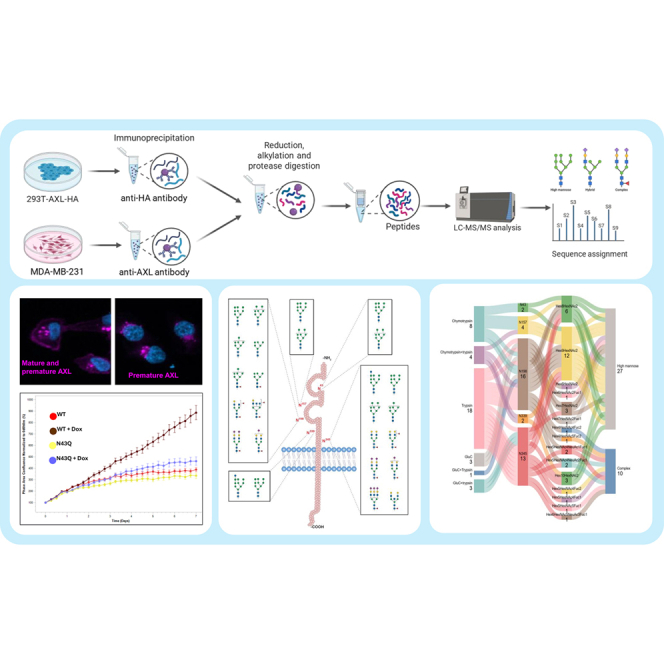

## In Brief

Site-specific glycoproteomics revealed that AXL receptor tyrosine kinase contains multiple N-glycosylation sites with diverse glycan structures. Glycan maturation is required for proper membrane trafficking, phosphorylation, and stability of AXL, and mutations at conserved sites N43 and N339 impair signaling and cell proliferation. These findings identify N-glycosylation as a key regulator of AXL oncogenic activity.

As an important member of the TAM (TYRO3, AXL, MER) receptor tyrosine kinase (RTK) family, AXL is a transmembrane protein that plays a pivotal role in regulating multiple cellular processes (1). AXL is typically activated by its ligand, Growth arrest-specific protein 6 (Gas6), which binds to the extracellular domain of AXL, triggering receptor dimerization and autophosphorylation on its intracellular domain (2, 3). Upon activation, AXL triggers downstream signaling through the PI3K–AKT, MAPK/ERK, and EGFR pathways, promoting cell survival, proliferation, and motility (4). Elevated expression of AXL, at both the mRNA and protein levels, is commonly observed across a variety of cancers, such as lung, breast, and pancreatic cancer (5, 6, 7, 8, 9, 10). High AXL expression is considered as a hallmark of epithelial–mesenchymal transition (EMT) and drives invasion and metastasis (11). AXL also confers therapeutic resistance by sustaining survival signaling when oncogenic drivers like EGFR or HER2 are inhibited (12). Beyond its intrinsic effects, AXL modulates the tumor microenvironment by promoting immune evasion and suppressing antitumor immunity (13). These immune effects reflect the fact that AXL signaling can impair the activation of immune cells, such as T-cells and natural killer (NK) cells (6, 14). AXL also correlates with stem-like phenotypes and tumor plasticity, enabling cells to adapt to stress and drug pressure (15). In melanoma cells, AXL acts downstream of WNT5A and regulates cancer cell dormancy (16). In addition to the full-length transmembrane form, a soluble isoform of AXL (sAXL), generated through proteolytic cleavage, has also been proposed as a marker of disease progression and poor survival, particularly in melanoma (17) and pancreatic ductal adenocarcinoma (18).

Given its multifaceted roles in tumor biology and immune evasion, AXL has emerged as a promising target for cancer therapies. Strategies aimed at blocking AXL activation or its interaction with Gas6 hold promises for suppressing tumor growth and enhancing the efficacy of immune-based treatments.

Glycosylation is the process in which a glycan is attached to a hydroxyl or other functional group of another molecule, forming a glycoconjugate (19). This modification can occur on proteins both co-translationally and post-translationally and is a ubiquitous process in eukaryotic cells. Glycosylation plays crucial structural and functional roles. Glycans not only affect the biological functions of normal cells but also play a vital role in the pathogenesis of cancer (20, 21). One of the most common forms of glycosylation is N-linked glycosylation, in which glycans are attached to the amide nitrogen of asparagine sidechains. The minimal canonical amino acid sequence for N-glycosylation begins with asparagine, followed by any amino acid except proline, and ends with serine or threonine (Asn-X-Ser/Thr) (22). N-glycosylation plays a crucial role in glycoprotein structure, stability, and function, including protein localization in membranes and interactions between proteins, such as ligands and receptors, particularly in membrane and secreted proteins (23, 24).

As an RTK, AXL is a glycoprotein mainly located on the cell membrane. However, the functional roles of N-linked glycosylation of human AXL in cancer remain poorly understood. In this study, we employed mass spectrometry-based proteomics and glycoproteomics to characterize the glycosylation profile of AXL and investigated how N-linked glycosylation affects its processing and function. To elucidate the functional role of AXL glycosylation, we employed enzymatic deglycosylation, small-molecule inhibition of glycosyltransferases, and site-directed mutagenesis of selected asparagine residues Our findings reveal that AXL exists in multiple glycoforms, predominantly bearing high mannose-type glycans and complex-type glycans. Additionally, AXL glycosylation plays a critical role in its membrane translocation, activation, and protein stability, and significantly influences AXL-mediated cell proliferation.

## Experimental Procedures

### Cell Culture

Human spontaneously immortalized breast epithelial cell line MCF10A, the embryonic kidney cell line HEK293T, and breast cancer cell lines MDA-MB-231, HCC1395, HCC1937, MCF7 and ovarian cancer cell line OV90 were obtained from the American Type Culture Collection (ATCC). Ovarian cancer cell lines HeyA8 and TYKnu were kindly provided by Ernst Lengyel (University of Chicago); and Ovcar8 cells were from Dominic Scudiero (NCI Frederick). All lines were confirmed by short tandem repeat profiling and assayed regularly for *mycoplasma*.

MCF10A cell lines (parental, wild-type AXL, and AXL mutant overexpression lines) were maintained in DMEM/F12 medium (Thermo Fisher Scientific; #11330-032) supplemented with 5% horse serum (Thermo Fisher Scientific; #16050-122), 20 ng/ml epidermal growth factor (Thermo Fisher Scientific; AF-100-15), 0.5 mg/ml hydrocortisone (Sigma; H0888), 100 ng/ml cholera toxin (Sigma; C8052), 10 μg/ml insulin (Sigma; I-1882), and 100 U/ml penicillin G–100 μg/ml streptomycin (Gibco; 15140-122). Epidermal growth factor (EGF) was omitted from the medium when doxycycline induction was started for both experimental and control MCF10A groups.

MDA-MB-231, HCC1395, HCC1937, MCF7, and HEK293T cells were cultured in RPMI-1640 medium supplemented with 10% fetal bovine serum (FBS; Gibco; 26140-079). OVCAR8, TYKnu, and OV90 cells were maintained in RPMI-1640 with 10% FBS and 2 mM glutamine. HeyA8 cells were cultured in high-glucose DMEM (Thermo Fisher Scientific; #11965092) with 10% FBS. All cell lines were maintained at 37 °C in a humidified atmosphere containing 5% CO_2_.

### Transient Transfection and Lentiviral Transduction

The inducible expression vector pLVX-TetOne-Puro-hAXL was obtained from Addgene (25). A HA-tag or an IRES-EGFP sequence was cloned in the downstream of the wild-type AXL coding sequence to generate pLVX-AXL-HA and pLVX-TetOne-hAXL-IRES-EGFP constructs. Site-directed mutagenesis of AXL (N43Q, N157Q, N198Q, N339Q, N345Q, N401Q) was performed using the Gibson Assembly Master Mix (NEB; E2611) (26, 27). Primer sequences are provided in Supplementary Table S1.

For transient expression, HEK293T cells were transfected with pLVX-AXL-HA using Lipofectamine 3000 and P3000 reagents. Lysates were collected 72 h post-transfection for AXL immunoprecipitation. Lentiviruses were produced in HEK293T cells via co-transfection of the pLVX-TetOne-Puro-hAXL plasmid (wild-type or mutant AXL), psPAX2, and pMD2.G packaging vectors. Viral supernatants were harvested post-transfection and used to transduce MCF10A, MDA-MB-231, and OV90 cells. Transduced cells were selected with 1 μg/ml puromycin for 1 week prior to subsequent experiments.

### Gene Overexpression and Knockdown

Overexpression of wild-type or glycosylation site-mutant AXL was induced with doxycycline (Sigma; D9891) for 72 h for Western blotting and for 7 days in proliferation assays. For siRNA-mediated knockdown, Ambion Silencer Select pre-designed AXL siRNAs (#S1845 and #S1847; Fisher Scientific) were transfected using Lipofectamine RNAiMAX (Invitrogen; #13778075). Cells were harvested 72 h post-transfection.

### Ligand, Inhibitor and Endoglycosidase Treatment

AXL activation was stimulated by GAS6 (R&D #885-GSB) at 400 ng/ml for 10 min. Complex N-glycan formation was inhibited by 10 μM kifunensine (Cayman #10009437) added 48 h prior to harvest. Deglycosylation of AXL was achieved by treating lysates with Endo H (NEB; P0702S) or PNGase F (NEB; P0704S) for 1 h at 37 °C. Cycloheximide (CHX) (Sigma; S7418) was applied at 50 μg/ml to block protein synthesis.

### Western Blot analysis

Cell lysate was obtained in a modified RIPA lysis buffer as described previously (28). Cell lysate containing 10 to 30 μg protein was subjected to SDS-PAGE and transferred to nitrocellulose membranes. Then, protein was detected by specific primary antibodies and horseradish peroxidase-conjugated secondary antibodies. The antibodies used in this study include: Anti-AXL (C89E7) (#8661, CST), phospho-AXL (Tyr702) (D12B2) (#5724, CST), β-actin (13E5) (#5125, CST), phospho-Akt (Ser473) (#4060, CST), AKT (pan) (C67E7) (#4691, CST), phospho-p44/42 MAPK (Erk1/2) (Thr202/Tyr204) (#4370, CST), p44/42 MAPK (Erk1/2) (#137F5, CST), HRP-conjugated anti-Rabbit IgG Antibody (#GENA934-1ML, Millipore), anti-mouse IgG Antibody (#GENA931-1ML, Millipore), and anti-goat IgG antibody (#A15999, Invitrogen). Rabbit monoclonal anti-AXL-pY703 and AXL-pY779 were generated by our lab.

### Immunoprecipitation for sAXL

Cells were switched to serum-free medium 12 h prior to harvest. The harvested culture medium was filtered through a 0.45 μm filter to remove cell debris and pre-cleared using protein G beads (#15920-010, Invitrogen) for 1 h. Next, 2 ml of the cell culture medium was incubated with 0.7 μg of human AXL antibody (#AF154, R&D) overnight at 4 °C, followed by the addition of 7 μl of Protein G beads for 1 h. The beads were then resuspended in 20 μl of sample buffer and mixed gently. To dissociate the immunocomplexes, the beads were boiled for 5 min. SDS-PAGE and immunoblotting were performed using anti-Axl antibody (#A302-167A, Bethyl Laboratories) to detect sAXL.

### Immunofluorescence Staining

Cells were cultured on 8-well chamber slides, fixed with paraformaldehyde, and then sequentially blotted with anti-AXL antibody (#AF154, R&D), anti-Goat-Alexa Fluor 647 (#A-21447, Invitrogen), and DAPI (#D1306, Invitrogen). Images were acquired on a Zeiss LSM980 confocal microscope.

### Cell Proliferation Assay

MCF10A cells harboring doxycycline-inducible wild-type or mutant AXL constructs were seeded at 1000 cells per well in 96-well plates, in triplicate, and subsequently induced with doxycycline. The wells were imaged every 6 h for 7 days, and cell confluence was measured on an IncuCyte SX5 analyzer (Sartorius).

### Immunoprecipitation and On-Beads Proteolytic Digestion for Glycopeptide Analysis

Cell lysates (1 mg protein per sample) from MDA-MB-231 and HEK293T-AXL-HA cells were subjected to immunoprecipitation. AXL–HA was pulled down using anti-HA agarose beads (26181, Thermo Fisher Scientific), while endogenous AXL was immunoprecipitated with anti-AXL antibody (#AF154, R&D) and protein G agarose (#15920-010; Invitrogen). Enriched AXL was reduced with dithiothreitol and alkylated with iodoacetamide and then digested using sequencing-grade trypsin (V5111; Promega), chymotrypsin (V1061, Promega), GluC (P8100S, NEB), or trypsin–chymotrypsin combinations.

### Mass Spectrometry Analysis

Peptides generated from on-bead digestion of immunoprecipitated AXL samples were analyzed by LC-MS/MS on an Orbitrap Eclipse, Ascend or Exploris 480 instrument. Previously published LC-MS/MS parameters (29) were used with some modifications. Briefly, peptides were separated by liquid chromatography on an EASY-Spray column (75 μm × 50 cm, PepMap RSLC C18, Thermo Fisher Scientific) packed with 2 μm C18 particles, maintained at 50 °C. We used 0.1% formic acid in water (solvent A) and 0.1% formic acid in acetonitrile (solvent B) as solvents. Peptides were trapped on a trap column (100 mm × 2 cm, Acclaim PepMap100 Nano-Trap, Thermo Fisher Scientific) at a flow rate of 20 μl/min. LC separation was performed at a flow rate of 300 nl/min, and the following gradient was used: equilibration at 3% solvent B from 0 to 4 min, 3% to 10% sol B from 4 to 10 min, 10% to 35% sol B from 10.1 to 125 min, 35% to 80% sol B from 125 to 145 min, followed by equilibration for next run at 5% sol B for 5 min. Experiments were done in data-dependent acquisition mode with top 15 ions isolated at a window of *m/z* 0.7 and default charge state of +2. Precursors with charge states ranging from +2 to +7 were considered for MS/MS events. Normalized stepped collision energy was applied to fragment precursors at energies of 15%, 25%, and 40%. Precursor ions were acquired in the Orbitrap mass analyzer in *m/z* range of 350–2000 for glycoproteomics and *m/z* 350–1800 for proteomics at a resolution of 120,000 (at *m/z* 200). Automatic gain control (AGC) for MS and MS/MS were 10^6^ and 1 × 10^5^, and injection times to reach AGC were 50 ms and 250 ms, respectively. The " exclude isotopes feature was set to “on,” and 60-s dynamic exclusion was applied. Data acquisition was performed with option of “lock mass” (*m/z* 441.12002) for all data. Raw data were searched against the human proteome.

### Database Searching and Data Analysis

Database searching for N-glycopeptides was performed using publicly available software pGlyco Version 3 (30). Human N-glycan databases already available with the software were used (Supplementary Table S3), and UniProt Human Reviewed protein sequences (UniProt release 2024_04, 20,435 entries, downloaded February 1, 2021) were used as protein sequence FASTA file. Respective enzyme-specificity was set for searching with 2 missed cleavages allowed, and precursor and fragment tolerance were set to 10 and 20 ppm, respectively. Carbamidomethylation of cysteine was set as fixed modification and deamidation of asparagine and glutamine were set as variable modifications. The results were filtered to retain entries that had <1% FDR at glycopeptide level. MS/MS spectra were manually verified for several glycan oxonium ions and quality. All sialic acid-containing glycopeptide spectra were verified for presence of sialic acid–specific oxonium ions at *m/z* 274.09, 292.10 and/or 657.23. Data were simultaneously searched using Sequest in Proteome Discoverer 3.0 to identify non-glycosylated peptides against the UniProt Human Reviewed protein sequences (UniProt release 2024_04, 20,435 entries). Respective enzyme-specificity was set for searching with 2 missed cleavages allowed, and precursor and fragment tolerance were set to 10 ppm and 0.02 Da, respectively. Carbamidomethylation of cysteine was set as fixed modification and deamidation of asparagine and glutamine were set as variable modifications. Methionine oxidation and protein N-terminal acetylation were also set as variable modifications. Target FDR (Strict) for peptides was set at 0.01.

To further increase confidence in glycopeptide identification, a secondary pGlyco search was performed using a customized protein database composed exclusively of proteins identified in the Proteome Discoverer analysis described above. Search parameters, glycan databases, enzyme specificity, mass tolerances, and FDR filtering criteria were identical to those used in the primary pGlyco search. For downstream analysis and reporting, only glycan compositions consistently identified in both the full UniProt and customized database searches were retained.

### Experimental Design and Statistical Rationale

All the biological experiments were repeated at least 3 times. All the data with statistical analysis has 3 to 6 technical replicates. Statistical analysis was performed using the GraphPad Prism software. Significant differences between groups were determined using the Mann-Whitney or one-way ANOVA with Tukey’s multiple comparison test. Statistical significance was defined as ∗*p* < 0.05, ∗∗*p* < 0.01, ∗∗∗*p* < 0.001, and *ns* for not significant.

## Result

### AXL is Glycosylated in Breast and Ovarian Cancer Cells

**Fig.1:**
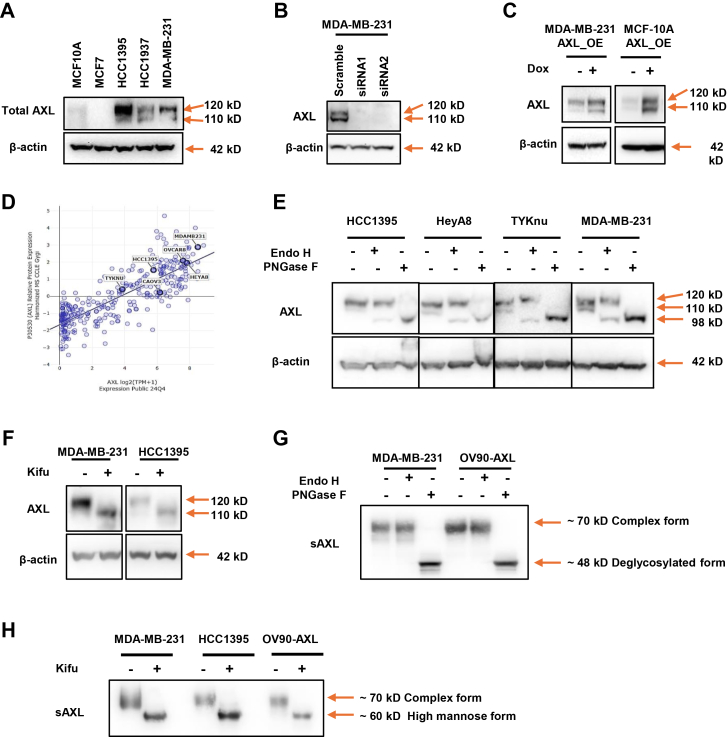
**Glycosylation of AXL in human cells.***A*, immunoblot analysis of AXL expression in breast cancer cell lines (HCC1395, HCC1937, MDA-MB-231, MCF7) and the non-tumorigenic breast epithelial cell line MCF10A. *B*, immunoblot showing AXL knockdown by two different AXL targeting siRNAs in MDA-MB-231 cells. *C*, immunoblot confirming the AXL inducible overexpression in MCF10A-AXL_OE and MDA-MB-231-AXL_OE cells. *D*, scatter plot showing AXL mRNA levels and protein expression levels among breast and ovarian cancer cell lines. Cancer cell lines selected for this study were labeled in the plot. *E*, immunoblot showing the electrophoretic mobility shift of AXL in HCC1395, HeyA8, TYKnu and MDA-MB-231 cell lysates treated with Endo H or PNGase F. *F*, immunoblot of AXL in MDA-MB-231 and HCC1395 cells treated with kifunensine. *G*. Soluble AXL (sAXL) was immunoprecipitated from MDA-MB-231 and OV90-AXL cell culture supernatants, treated with PNGase F or Endo H and immunoblotted with anti-AXL antibody. *H*. sAXL was immunoprecipitated from the cell culture supernatants of MDA-MB-231, HCC1395, and OV90-AXL cells, treated with kifunensine and immunoblotted with anti-AXL antibody. b-actin served as loading control in all immunoblots.

AXL protein consists of 894 amino acids, with an expected molecular weight of approximately 98 kD. However, immunoblot analysis of the breast cancer cell lines HCC1395, HCC1937, MDA-MB-231, and a normal breast epithelial cell line MCF10A revealed two predominant AXL bands at ∼ 120 kD and ∼110 kD (Fig. 1*A*). To confirm that both bands correspond to AXL, we performed siRNA-mediated AXL knockdown in MDA-MB-231 cells, which resulted in the loss of both bands (Fig. 1*B*). To further rule out the possibility that the two bands are the result of alternative splicing, we generated doxycycline-inducible AXL-overexpressing cell lines (MCF10A-AXL_OE and MDA-MB-231-AXL_OE) using the AXL sequence (NM_021913) coding the longest isoform of AXL. Upon doxycycline induction, both bands were markedly intensified, indicating that the observed molecular weight shifted from 98 KD to 120 kD and 110 kD, and these two forms of AXL are likely due to different post-translation modifications such as glycosylation (Fig. 1*C*). To investigate whether the glycans attached to AXL cause the molecular shift and generate the two different AXL molecular weight forms, we selected several AXL-expressing breast and ovarian cancer cell lines with medium to high AXL expression levels (Fig. 1*D*) and treated the cell lysates with or without endoglycosidases Endo H or PNGase F (Fig. 1*E*). Immunoblot analysis of these cell lysates without endoglycosidase treatment consistently showed an upper band (∼120 kD) and a lower band (∼110 kD). Endoglycosidase digestion revealed that the lower band was sensitive to both Endo H and PNGase F, whereas the upper band was only sensitive to PNGase F (Fig. 1*E*). Since PNGase F removes all N-glycans, while Endo H specifically cleaves high-mannose and some hybrid-type N-glycans, these results suggest that the 110 kD band represents AXL modified with high-mannose N-glycans, the 120 kD band corresponds to AXL carrying complex N-glycans, and the 98 kD band after PNGase F digestion corresponds to the core polypeptide of AXL protein without glycans. To validate this hypothesis, we treated the cell lines with kifunensine, a small-molecule inhibitor of mannosidase I, to block the maturation of high-mannose N-glycans on the AXL protein (Fig. 1*F*). Following the treatment, all detected AXL proteins accumulated at ∼ 110 kD, confirming that both observed bands represent N-glycosylated isoforms of AXL and that the 110 kD form corresponds to AXL modified with high-mannose type N-glycans.

The juxtamembrane region of AXL can be cleaved by metalloproteinases, such as ADAM10 and ADAM17, generating a soluble form of AXL (sAXL) (31, 32). Several studies have reported that N-glycosylation regulates ectodomain shedding (33, 34). To determine whether both glycosylated forms of full-length AXL undergo cleavage to produce sAXL and whether they are involved in ectodomain shedding of AXL, culture supernatants from the breast cancer cell line MDA-MB-231 cells were subjected to immunoprecipitation using an antibody recognizing the extracellular domain of AXL, followed by digestion with endoglycosidases Endo H and PNGase F (Fig. 1*G*). We also generated an AXL overexpression cell line OV90-AXL using ovarian cancer cell line OV90, which lacks detectable endogenous AXL, and analyzed its culture supernatants. We found that glycosylated sAXL was sensitive to PNGase F but not Endo H in both MDA-MB-231 and OV90-AXL cells, indicating that the predominant form of sAXL carries complex N-glycans. Because full-length AXL contains both high-mannose and complex N-glycans, this result suggests that glycosylation-matured AXL is preferentially shed by metalloproteinases. Interestingly, inhibiting complex glycan maturation with kifunensine did not block AXL ectodomain shedding (Fig. 1*H*).

Taken together, both sAXL and full-length AXL are glycosylated and carry complex or high mannose glycans, indicating a possible role for glycosylation in AXL biogenesis and function.

### Glycosylation is Essential for AXL Membrane Translocation and Phosphorylation

**Fig. 2:**
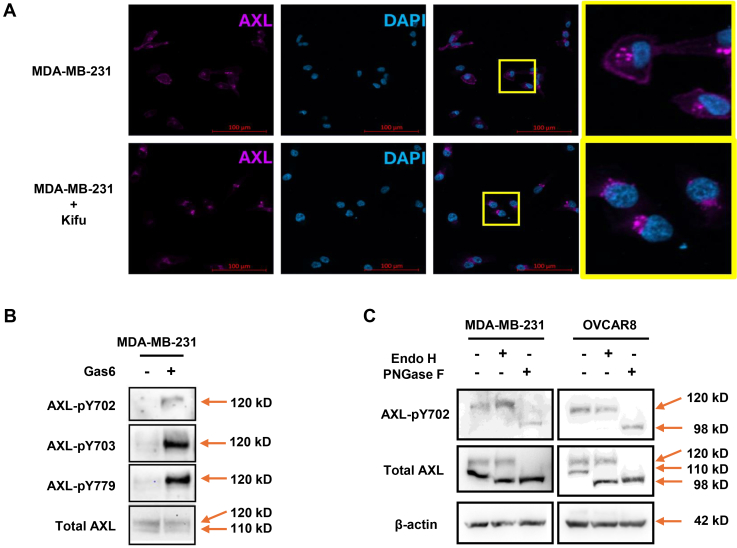
**Glycosylation of AXL is essential for its membrane localization and phosphorylation.***A*, immunofluorescence staining of AXL (*purple*) in MDA-MB-231 cells treated with or without kifunensine. DAPI staining was applied to visualize nuclei. *B*, immunoblot to examine AXL phosphorylation at indicated sites and AXL protein expression in MDA-MB-231 cells treated with or without GAS6. *C*. Immunoblot of AXL phosphorylation and AXL expression in MDA-MB-231 and OVCAR8 cell lysates treated with PNGase F or Endo H. b-actin served as loading control.

Previous studies have shown that glycosylation deficiency can alter the subcellular localization of proteins (24, 35, 36, 37). N-linked glycosylation begins in the endoplasmic reticulum (ER), where a glycan precursor is attached to an asparagine (Asn, N) residue and trimmed by glucosidases, forming high-mannose N-glycans. Glycoproteins are then transported from the ER to the Golgi apparatus, where mannosidases remove mannose residues and glycosyltransferases modify the glycans by addition of other monosaccharides such as N-acetylglucosamine, galactose, sialic acid and fucose, converting them into complex N-glycans. As glycan maturation progresses, some membrane proteins translocate to the cell surface (38, 39). In this study, by blocking N-glycan biosynthesis at the high mannose glycan trimming stage using kifunensine, a small-molecule inhibitor of mannosidase I, we found that more immature AXL accumulated in the cytoplasm than located on the cell membrane (Fig. 2*A*).

As a receptor tyrosine kinase, AXL is activated by its ligand GAS6, leading to the phosphorylation of specific tyrosine residues within its intracellular domain, which regulates downstream signaling (40, 41, 42). To investigate the role of AXL glycosylation, we stimulated MDA-MB-231 cells with GAS6 to activate AXL and assessed its phosphorylation using Western blot and immunoprecipitation. Notably, almost all the phosphorylated AXL was detected as complex glycosylated forms (Fig. 2*B*), with identical results observed across all three tyrosine phosphorylation sites. This finding was further confirmed by Endo H and PNGase F digestion in breast cancer cell line MDA-MB-231 and the ovarian cancer cell line OVCAR8 (Fig. 2*C*), with PNGase F digestion causing a decrease in size of phosphorylated AXL, demonstrating that AXL phosphorylation mainly occurs on its complex glycosylated form.

### Identification of Glycosylation Sites in AXL Protein

**Fig. 3:**
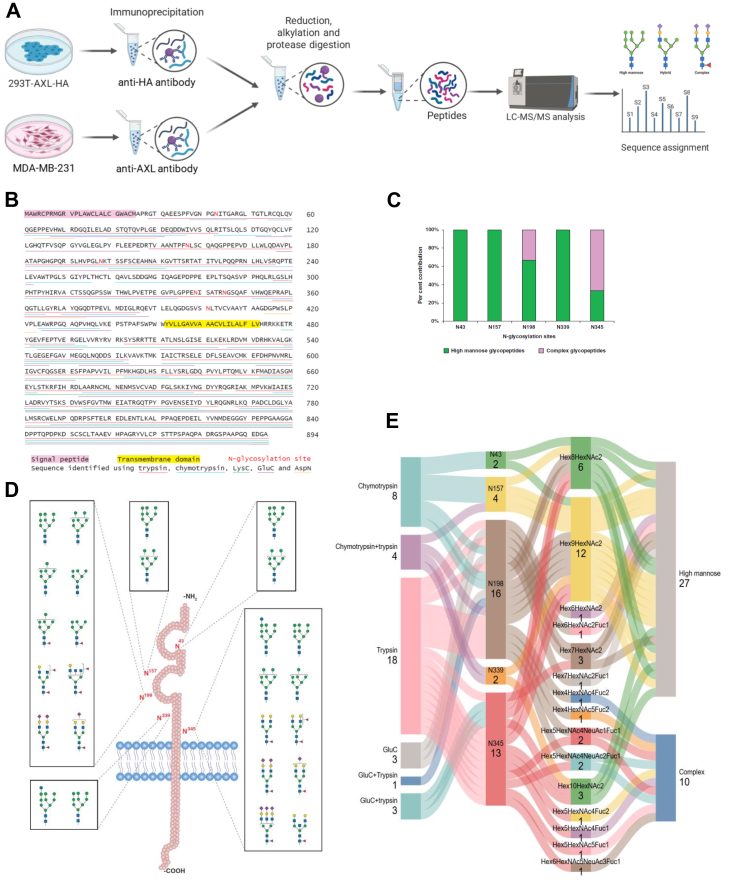
**Site-specific N-glycosylation profiling of AXL.***A*, a Schematic workflow showing AXL glycoproteomic analysis. *B*, full-length amino acid sequence of AXL showing experimentally identified peptides and annotated N-glycosylation sites (N43, N157, N198, N339, N345, and N401). The signal peptide, transmembrane domain, and N-glycosylation sites were highlighted. *C*, quantitative distribution of high-mannose and complex glycans at individual N-glycosylation sites. *D*, schematic representation of AXL structure and corresponding glycosylation sites. Unique glycans detected at each site are illustrated, with high-mannose and complex N-glycans shown. Putative structures are shown using Symbol Nomenclature for Glycans (52) and represent glycan composition inferred from mass spectrometry data. *E*, sankey diagram mapping N-glycans identified on AXL across different protease digestions. The diagram shows the distribution of glycopeptides produced by various protease treatments across AXL N-glycosylation sites and their associated high-mannose or complex N-glycan structures.

**Fig. 4:**
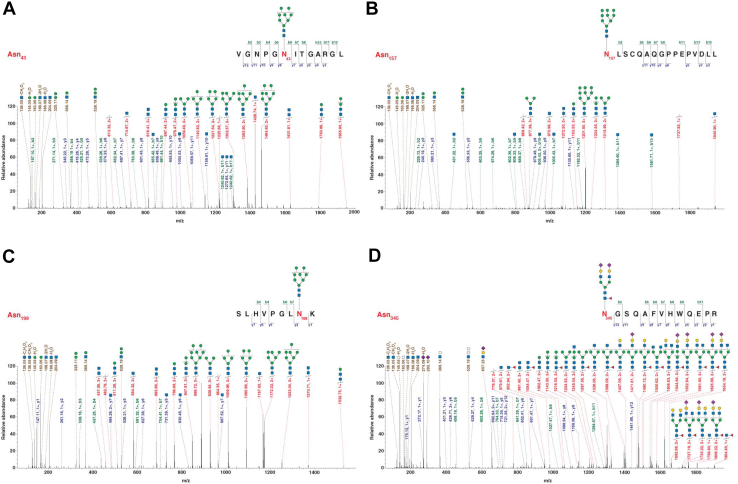
**Spectra of AXL N-glycopeptides.** (*A–D*) Representative spectra of glycopeptides corresponding to AXL N-glycosylation sites N43 (*A*), N157 (*B*), N198 (*C*), and N345 (*D*), showing diagnostic peptide backbone and glycan fragment ions. Putative glycan structures are annotated according to the Symbol Nomenclature for Glycans (52) and reflect compositions inferred from mass spectrometry data.

Given the pivotal role of phosphorylation in AXL function and the profound impact of glycosylation on AXL phosphorylation, we aimed to identify and characterize the N-linked glycosylation sites on AXL using a glycoproteomic approach (Fig. 3*A*). To enrich the AXL protein, we employed both MDA-MB-231 cells, with high expression level of endogenous AXL, and 293T-AXL-HA cells, with overexpressed HA-tagged AXL. AXL proteins were immunoprecipitated by anti-AXL antibody or anti-HA agarose, followed by digestion with different endoproteinases, including trypsin, chymotrypsin, LysC, AspN or GluC separately, or with sequential digestion using trypsin and chymotrypsin or trypsin and GluC. To identify N-linked glycosylation events in a site-specific manner, we performed bottom-up mass spectrometry-based glycoproteomics analysis on the enriched samples. As shown in Figure 3*B*, AXL protein was identified with excellent sequence coverage (93.9%, 797/848 excluding the signal peptides and the transmembrane domain) by deploying protease digestion with multiple enzymes separately and in combination. A total of thirty-three N-glycopeptides were identified from AXL with glycosylation at sites N43, N157, N198, N339 and N345. Monosaccharide composition of the identified glycopeptides indicates that AXL is glycosylated with considerable microheterogeneity at the identified sites with both high mannose as well as complex glycans with variable sialylation and fucosylation (Fig. 3*C*). Interestingly, glycopeptides with fragmentation signatures of bisecting GlcNAc were identified at N345. Distribution of these glycopeptide types by site is shown in Figure 3, *D* and *E*. Representative annotated MS/MS spectra showing evidence of glycosylation at the five identified sites are shown in Figure 4, *A*–*D* and Supplementary Figure.

A complete list of identified glycopeptides is provided in Figure 3*E* and Supplementary Table S2, in which the five N-glycosylation sites, N43, N157, N198, N339, and N345, were broadly categorized based on the complexity of their attached glycans. Sites N43, N157, and N339 were identified with high-mannose structures (specifically Hex8HexNAc2 and Hex9HexNAc2), indicating they are either less accessible to processing enzymes in the Golgi or that the AXL population carrying glycans at these positions is rapidly shuttled out of the complex glycan synthesis machinery (43, 44, 45). In this study, N43 and N157 were found to exclusively bear high-mannose structures (2 structures), while N339 was also predominantly high-mannose (2 unique structures, with one of them being a processing intermediate with a glucose residue still attached). In contrast, N198 and N345 showed a significant level of heterogeneity, featuring both high-mannose and more processed complex glycans. This suggests they are highly accessible to the full enzymatic machinery of the Golgi. N198 exhibited the greatest diversity, showing both complex (4 structures) to high-mannose (6 structures) glycans, with complex forms including various fucosylation and sialylation modifications (e.g., Hex5HexNAc4NeuAc1Fuc1). N345 also displayed a rich mixture of glycan types, with a slightly higher proportion of complex structures (6 structures) compared to high-mannose forms (4 structures). Importantly, glycans with composition and fragmentation pattern consistent with a bisecting GlcNAc modification were identified at site N345.

### The Role of N-Glycosylation in AXL Function, Cellular Trafficking, and Stability

**Fig. 5:**
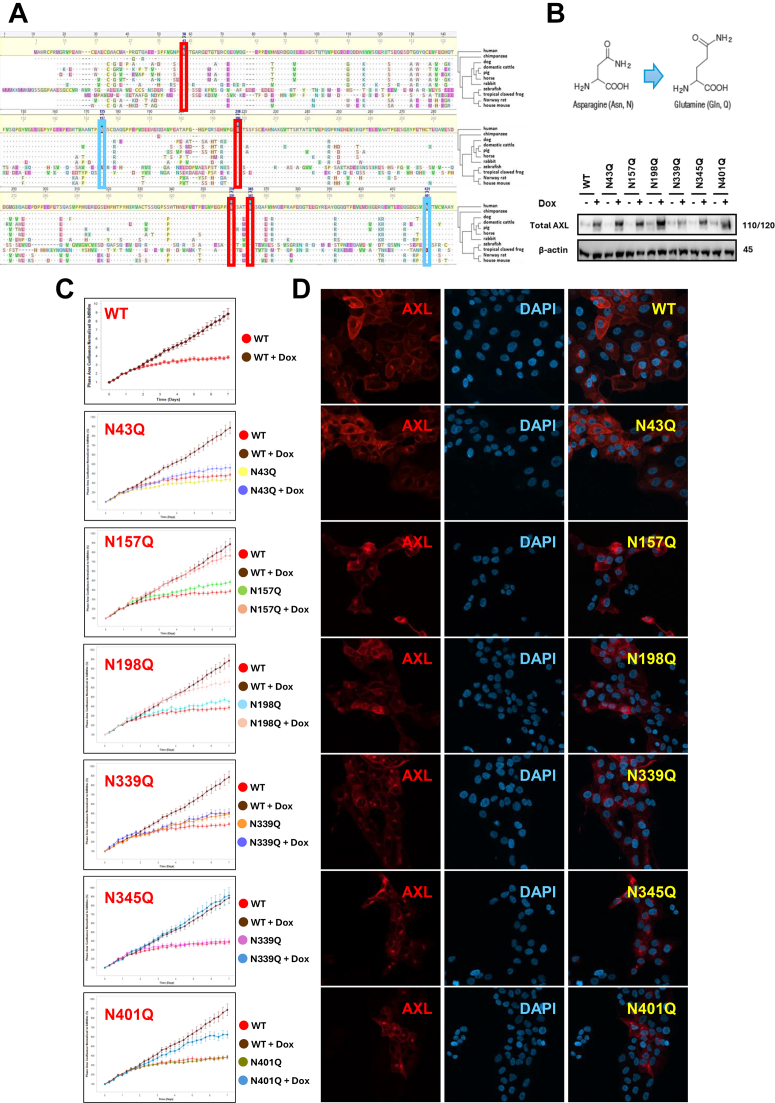
**Functional analysis of AXL glycosylation.***A*, multiple sequence alignment and phylogenetic tree analysis of AXL protein sequences from eleven vertebrate animals, including humans. Rectangle boxes indicate the six reported glycosylation sites of AXL. Most conserved sites are in red, and relatively less conserved sites are in blue. *B*, immunoblot to examine AXL expression in MCF10A cells transduced with AXL-WT and single-site glycosylation-deficient AXL (as indicated). β-actin serves as a loading control in all immunoblots. *C*, cell proliferation curves of MCF10A cells expressing doxycycline-inducible wild-type (WT) or individual AXL N-glycosylation site mutants (N43Q, N157Q, N198Q, N339Q, N345Q, and N401Q). Cell growth was monitored over time in triplicate cultures using live-cell imaging in IncuCyte. Data represent mean ± s.d. *D*, immunofluorescence staining of AXL (*red*) and nuclei (DAPI, *blue*) in MCF10A cells expressing WT or mutated AXL. WT AXL showed predominant membrane localization, whereas glycosylation-site mutants exhibited reduced membrane expression and altered subcellular distribution.

To assess the functional importance of these glycosylation sites in AXL, we first examined the AXL protein sequence conservation by comparing the human AXL sequence with those of 10 other vertebrate species and constructed a phylogenetic tree based on AXL protein conservation (Fig. 5*A*). Here, considering the potential heterogeneity of glycosylation across different cell types, all six reported sites were included in our analyses, including N401, which was not detected in our mass spectrometry analyses. Amino acid sequence alignment revealed that four out of the six glycosylation sites (N43, N198, N339, and N345) are highly conserved, whereas two sites (N157 and N401) exhibit relatively lower conservation, with a single amino acid variant observed among the eleven species.

To investigate the role of N-glycosylation in AXL function and cellular trafficking, we generated inducible expression vectors to produce both wild-type and single-site glycosylation-deficient AXL variants by mutating each of the six asparagine residues with glycosylation in its extracellular domain into glutamine residues. These AXL coding constructs were packaged into lentiviruses and used to infect the MCF10A breast epithelial cell line, which has low intrinsic AXL expression. Upon doxycycline induction, single-site glycosylation-deficient AXL proteins were expressed in the targeted cells, whereas only minimal AXL expression was detected in the corresponding non-induced cells (Fig. 5*B*). Immunoblot analysis also revealed that all single-site glycosylation-deficient variants were still glycosylated, indicated by no observed major molecular weight shift.

MCF10A cells were derived from spontaneously immortalized mammary gland epithelial cells, and the proliferation of MCF10A cells relies on the presence of epidermal growth factor (EGF). When EGF was removed, MCF10A cells grew very slowly, while overexpression of wild-type AXL compensated for the function of EGF and significantly enhanced the proliferation of MCF10A cells in EGF-free medium (Fig. 5*C*). Notably, the proliferation-promoting effect was markedly reduced in cells harboring the N43Q and N339Q mutations, while a moderate decrease was observed in the N157Q, N198Q, and N401Q mutants. In contrast, the N345Q mutation had little to no impact compared to wild-type AXL. These findings suggest that evolutionarily conserved N43 and N339 are critical glycosylation sites for AXL-mediated cell proliferation.

We further examined AXL subcellular localization via immunofluorescence staining using an AXL antibody. Wild-type AXL was predominantly localized on the cell membrane, with a small fraction accumulated in the perinuclear cytoplasm (Fig. 5*C*). In single-site mutants, slightly increased cytoplasmic retention of AXL was observed (Fig. 5*C*). Given that inhibiting N-glycosylation maturation of AXL with kifunensine substantially impaired AXL membrane localization (Fig. 2*A*), these results suggest that N-glycosylation is essential for AXL membrane translocation, with multiple glycosylation sites collectively contributing to this process.

**Fig. 6:**
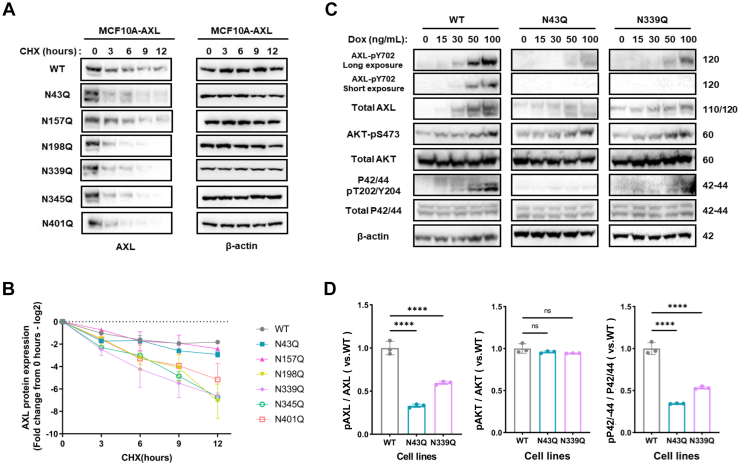
**N-linked glycosylation sites stabilize AXL protein and are critical for its downstream signaling.***A*, AXL protein levels were assessed in MCF10A-AXL cells expressing either the WT or glycosylation sites mutated AXL over a time-course CHX treatment. AXL level was detected by Western blot, and β-actin served as a loading control. *B*, degradation rate of wild-type and glycosylation site deficient AXL variants. Data represents mean value of the densitometry analysis of AXL level normalized to β-actin and expressed relative to the 0-h time point for each cell line, from three independent experiments. *C*, MCF10A-AXL cells expressing WT, N43Q, and N339Q AXL were treated with increasing concentrations of doxycycline (0–100 ng/ml) for 24 h to induce AXL expression. Phosphorylated and total AXL (pY702), AKT (pS473), and p42/44 MAPK (pT202/Y204) were detected by Western blot. β-actin served as a loading control. *D*. Densitometry analysis to quantify AXL, AKT, and p42/44 phosphorylation at maximal induction (100 ng/ml Dox). Phosphorylation signals were normalized to total protein levels and expressed relative to WT AXL. Data represent mean ± s.e.m. from three independent experiments. ∗∗∗∗*p* < 0.0001; ns, not significant).

Because glycosylation is crucial in regulating stability of other proteins (36, 46, 47), we asked whether the functional changes seen in Figure 5*D* were partially due to a change in AXL stability. To answer this question, we examined AXL protein stability using cycloheximide treatment to inhibit protein synthesis, followed by Western blot analysis. All six single-site mutations resulted in reduced AXL stability, with over 90% degradation observed in the N198Q, N339Q, N345Q, and N401Q mutants and moderate reductions in the N43Q and N157Q mutants after 12-h of cycloheximide treatment, compared with wild-type AXL (Fig. 6, *A* and *B*). These results suggest that glycosylation plays a crucial role in maintaining AXL stability.

Given the critical role of AXL phosphorylation in downstream signaling (45, 46, 47) and the observation that most phosphorylated AXL carries complex-type glycans (Fig. 2*C*), we sought to identify which glycosylation sites are essential for AXL activation. Because the N43Q and N339Q mutations showed the strongest inhibition on cell proliferation (Fig. 5*D*), subsequent analyses focused on these two sites. Immunoblotting revealed that AXL phosphorylation increased with higher doxycycline concentrations, consistent with concentration-dependent receptor dimerization and autoactivation. However, this autoactivation was markedly diminished in the N43Q and N339Q mutants (Fig. 6, *C* and *D*). These findings, together with the reduced proliferation caused by the two mutants (Fig. 5*C*), indicate that glycosylation at the conserved N43 and N339 sites is critical for regulating AXL activation. Analysis of downstream pathways further showed that although both wild-type and mutant AXL could activate AKT, ERK phosphorylation at T202/Y204 was substantially reduced in the mutants (Fig. 6, *C* and *D*), suggesting impaired MAPK pathway activation.

Overall, our findings demonstrate that N-glycosylation is essential for AXL function, regulating its phosphorylation, stability, cellular localization, and proliferative capacity. In particular, N43 and N339 emerge as critical glycosylation sites required for AXL-mediated phosphorylation and cell proliferation, underscoring the central role of glycosylation in AXL regulation.

## Discussion

Receptor Tyrosine Kinases (RTKs) are vital cell surface receptors that regulate essential cellular processes like cell growth, survival, and metabolism. These receptors are activated when they bind to specific ligands, triggering phosphorylation of tyrosine residues and initiating downstream signaling. Dysregulated RTK signaling is implicated in various diseases, including cancer, and post-translational modifications such as glycosylation can significantly impact RTK function (23, 48). In this study, we focused on AXL, an RTK involved in cancer progression, and explored the role of glycosylation in its activity, trafficking, and stability.

We found that AXL is glycosylated in breast and ovarian cancer cells, as evidenced by the presence of two major isoforms at approximately 120 kD and 110 kD in MCF10A and MDA-MB-231 cells. These bands represent different glycosylated forms of AXL, with the 120 kD form carrying complex N-glycans and the 110 kD form modified with high-mannose N-glycans. This was confirmed by enzymatic digestion using endoglycosidases, which revealed distinct glycosylation patterns. These findings suggest that AXL undergoes differential glycosylation, and the types of glycans attached to the protein are crucial for its function.

We also explored how glycosylation affects the generation of sAXL, a cleaved form of AXL that hold the promise of to be developed as a novel non-invasive biomarker for multiple cancers and inflammatory diseases (17, 18, 40, 49, 50). Our results showed that sAXL predominantly carries complex N-glycans, suggesting that the modification of AXL with complex glycans might be important for its full functionality. However, blocking high-mannose glycan maturation using kifunensine did not prevent sAXL production, suggesting that complex glycosylation is not required for AXL cleavage and release.

An important finding in this study was the role of glycosylation in AXL’s membrane translocation and phosphorylation. When glycan biosynthesis was inhibited by kifunensine, AXL accumulated in the cytoplasm, indicating that glycosylation is essential for its proper trafficking to the cell membrane. Intriguingly, we also found that AXL phosphorylation, critical for its activation, was associated with its complex glycosylated form. Inhibition of glycan maturation resulted in reduced phosphorylation, highlighting the importance of glycosylation in facilitating AXL activation.

To further elucidate the specific glycosylation sites contributing to AXL function, we conducted mass spectrometry–based glycoproteomic analyses. Although six N-glycosylation sites of AXL are annotated in UniProt, only five major N-glycosylation sites were identified in the breast cancer cell line MDA-MB-231 and in the AXL-overexpressing 293T-AXL cell line. Given the potential heterogeneity of glycosylation across different cell types, all six reported sites were included in subsequent analyses. Phylogenetic analysis of AXL protein sequences from eleven vertebrate species revealed that four sites (N43, N198, N339, and N345) are highly conserved, suggesting their evolutionary and functional significance. To determine the functional relevance of these glycosylation sites, we generated single-site glycosylation-deficient AXL mutants. Notably, mutations at the highly conserved N43 and N339 sites resulted in markedly reduced AXL-mediated cell proliferation and phosphorylation, highlighting the essential role of these glycosylation sites in regulating AXL activity.

Additionally, our study highlighted the role of glycosylation in AXL protein stability. In cells expressing glycosylation-deficient mutants, AXL was less stable, with certain mutants undergoing significant degradation. This suggests that glycosylation protects AXL from degradation, which is particularly important in cancer where protein stability is often dysregulated.

In conclusion, our study demonstrates that N-glycosylation plays a crucial role in regulating AXL’s function through effects on cellular localization, phosphorylation, and stability. We identified key glycosylation sites, such as N43 and N339, that are essential for AXL’s activity. These findings offer valuable insights into AXL regulation and suggest that targeting glycosylation could provide a potential therapeutic approach for modulating AXL function in cancer.

## Data Availability

The glycoproteomic data has been deposited to the ProteomeXchange Consortium via the PRIDE (51) partner repository with the dataset identifiers PXD071027. Reviewers can access the dataset by logging in to the PRIDE website using the following account details:

Username: reviewer_pxd071027@ebi.ac.uk

Password: FCxw557L2saj

## Supplemental data

This article contains supplemental data.

## Conflict of Interest

The authors declare that they have no conflicts of interest with the contents of this article.
