# Supplementary figures and images for: N-Glycosylation of AXL Receptor Tyrosine Kinase Regulates Its Stability, Phosphorylation, and Oncogenic Function
Source: Mol Cell Proteomics. 2026 Apr 27;25(6):101574. doi: 10.1016/j.mcpro.2026.101574 (PMC13240818; doi:10.1016/j.mcpro.2026.101574)

Relative abundance

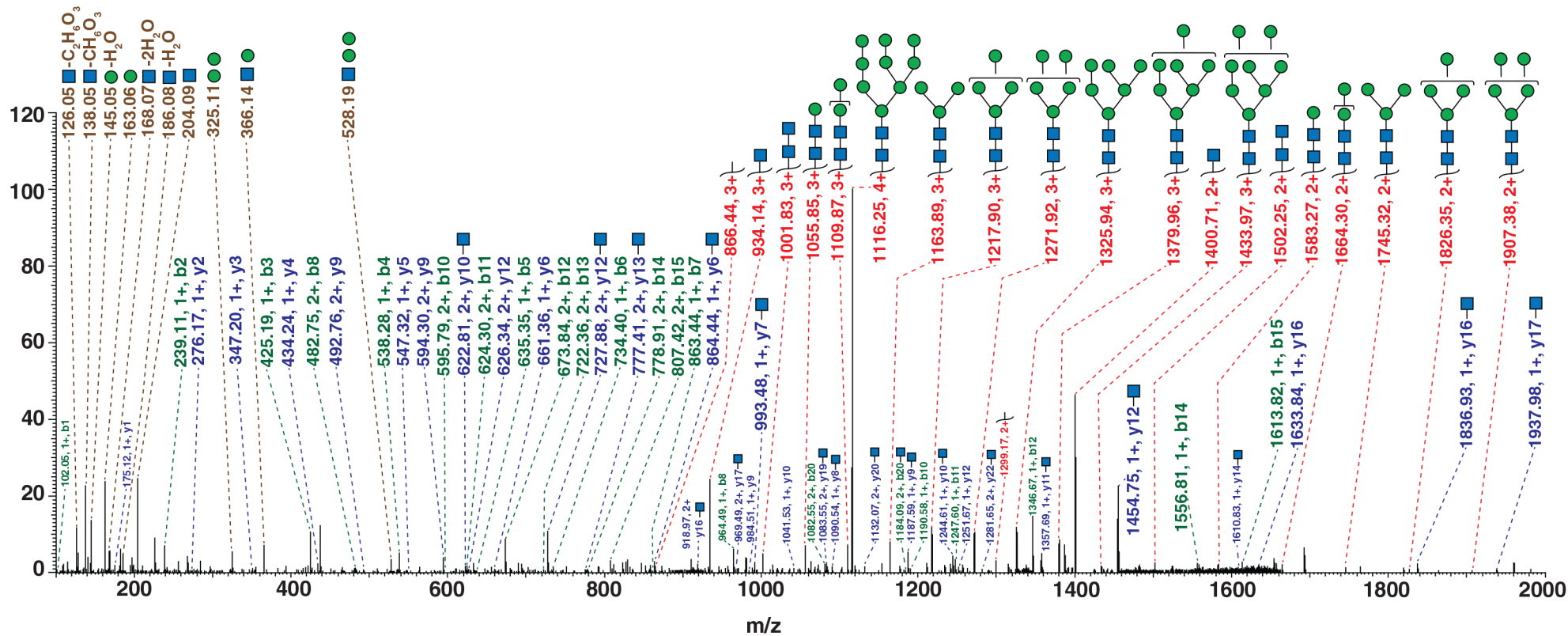

Supplement: Supplemental Figure S1 [file mmc4.pdf]
