# Supplementary material for: N-Glycosylation of AXL Receptor Tyrosine Kinase Regulates Its Stability, Phosphorylation, and Oncogenic Function
Source: Mol Cell Proteomics. 2026 Apr 27;25(6):101574. doi: 10.1016/j.mcpro.2026.101574 (PMC13240818; doi:10.1016/j.mcpro.2026.101574)
Supplement: Supplemental Figure S2 [file mmc5.pdf]

Supplementary data

Wang et al., N-glycosylation of AXL receptor tyrosine kinase regulates its stability, phosphorylation and oncogenic function, 2026

Annotated MS/MS spectra for AXL-derived glycopeptides generated using gLabel in pGlyco 3

Legend:

- J: Asn (N) in N-glycosylation motif
- Hexose
- N-Acetylhexosamine
- N-acetylneuraminic acid
- Fucose

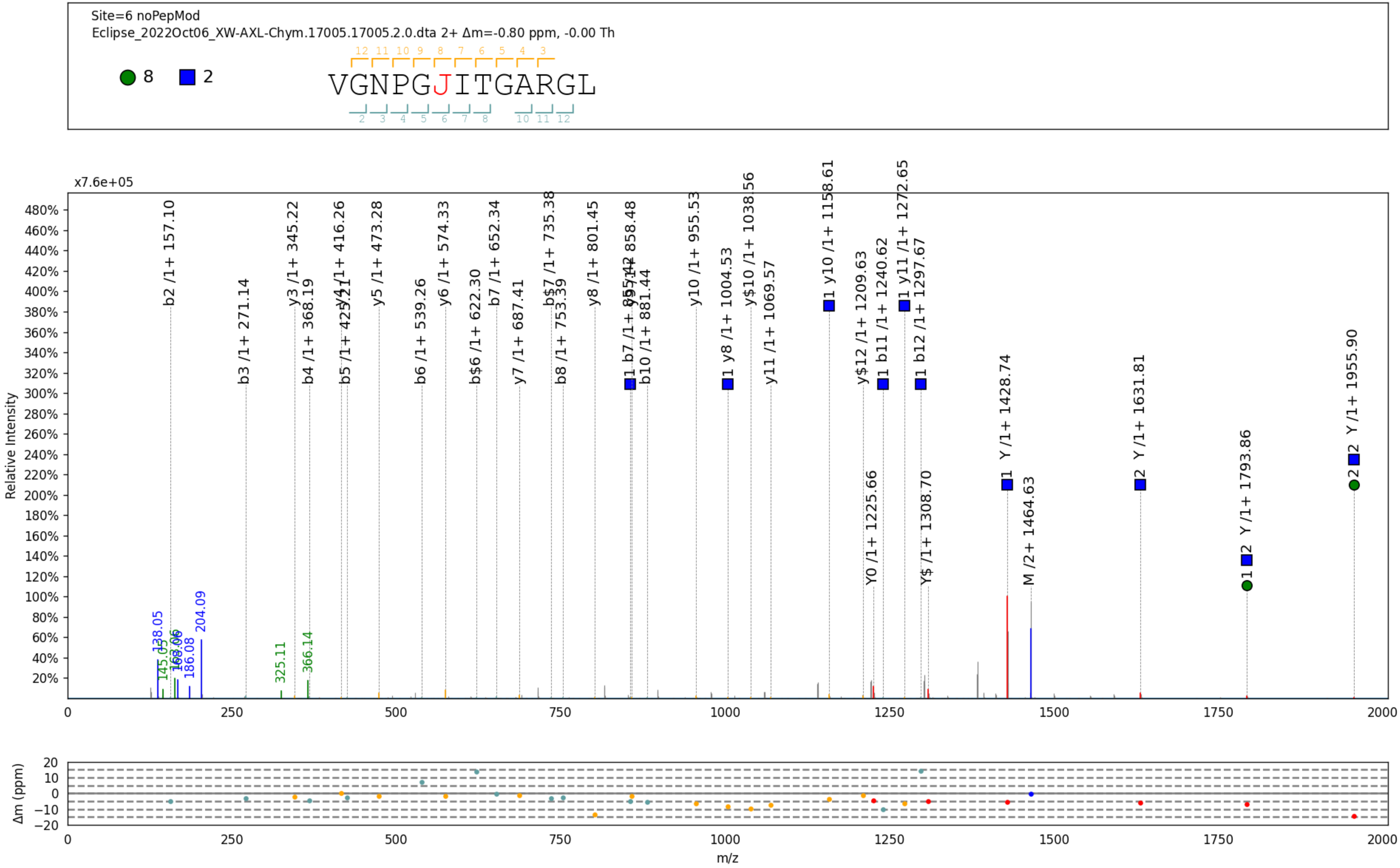
Site=6 noPepMod
Eclipse_2022Oct06_XW-AXL-Chym.17005.17005.2.0.dta 2+ Δm=-0.80 ppm, -0.00 Th
8
2
VGNPGJITGARGL
x7.6e+05
Relative Intensity
m/z
Δm (ppm)

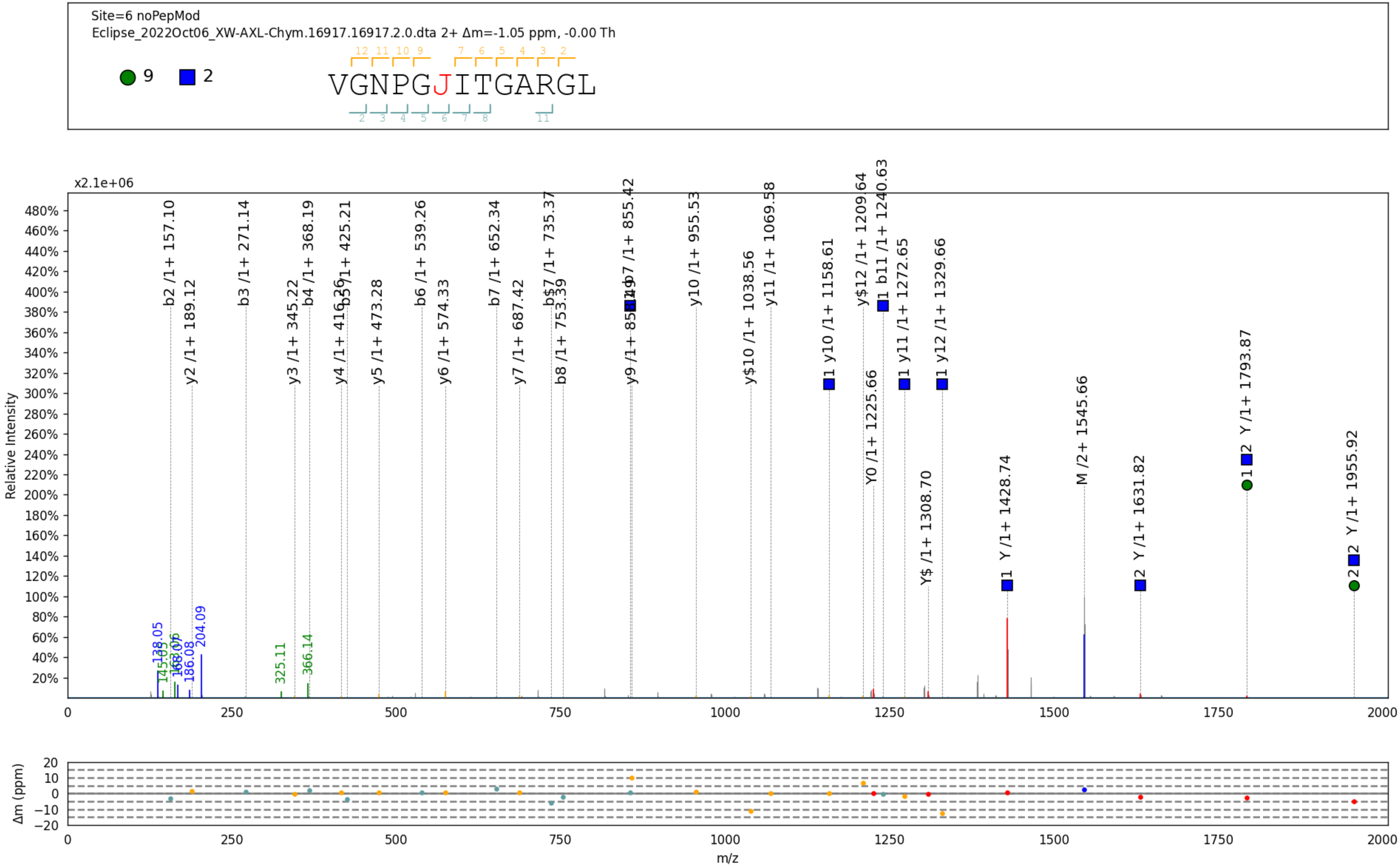

Site=6 noPepMod
Eclipse_2022Oct06_XW-AXL-Chym.16917.16917.2.0.dta 2+ Δm=-1.05 ppm, -0.00 Th
9
2
VGNPGJITGARGL
x2.1e+06
Relative Intensity
Δm (ppm)
m/z
b2 /1+ 157.10
y2 /1+ 189.12
b3 /1+ 271.14
y3 /1+ 345.22
b4 /1+ 368.19
y4 /1+ 416.26
b5 /1+ 425.21
y5 /1+ 473.28
b6 /1+ 539.26
y6 /1+ 574.33
b7 /1+ 652.34
y7 /1+ 687.42
b$7 /1+ 735.37
b8 /1+ 753.39
y9 /1+ 858.49
y10 /1+ 955.53
y$10 /1+ 1038.56
y11 /1+ 1069.58
1 y10 /1+ 1158.61
y$12 /1+ 1209.64
Y0 /1+ 1225.66
1 b11 /1+ 1240.63
1 y11 /1+ 1272.65
Y$ /1+ 1308.70
1 y12 /1+ 1329.66
1 Y /1+ 1428.74
M /2+ 1545.66
2 Y /1+ 1631.82
1 2 Y /1+ 1793.87
2 2 Y /1+ 1955.92
138.05
145.05
163.06
168.07
186.08
204.09
325.11
366.14

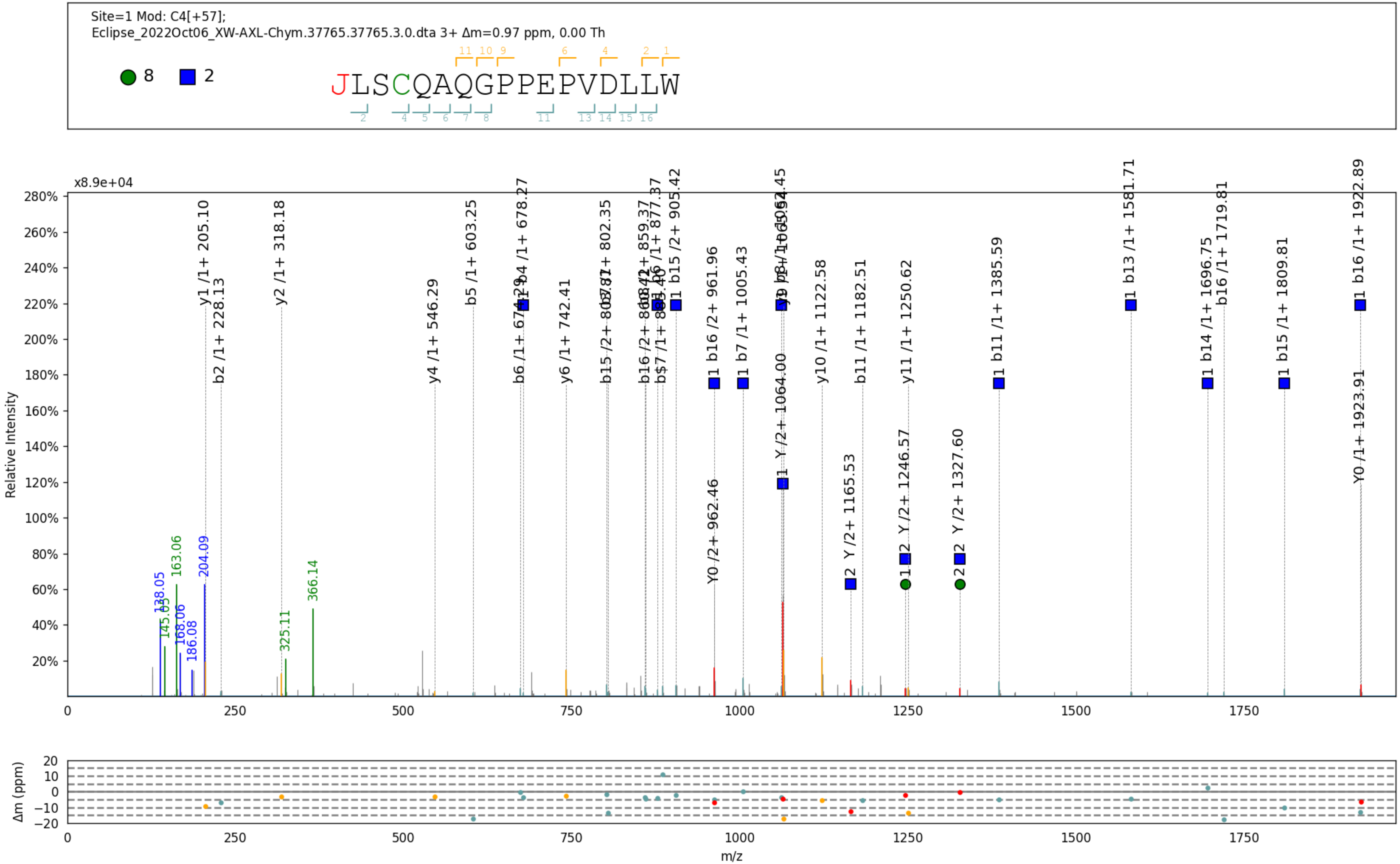

Site=1 Mod: C4[+57];
Eclipse_2022Oct06_XW-AXL-Chym.37765.37765.3.0.dta 3+ Δm=0.97 ppm, 0.00 Th
8
2
JLSCQAQGPPEPVDLLW
x8.9e+04
Relative Intensity
y1 /1+ 205.10
b2 /1+ 228.13
y2 /1+ 318.18
y4 /1+ 546.29
b5 /1+ 603.25
b6 /1+ 678.27
y6 /1+ 742.41
b15 /2+ 802.35
b16 /2+ 859.37
b6 /1+ 877.37
1 b15 /2+ 905.42
Y0 /2+ 962.46
1 b16 /2+ 961.96
1 b7 /1+ 1005.43
1 Y /2+ 1064.00
y10 /1+ 1122.58
b11 /1+ 1182.51
y11 /1+ 1250.62
2 Y /2+ 1165.53
1 2 Y /2+ 1246.57
2 2 Y /2+ 1327.60
1 b11 /1+ 1385.59
1 b13 /1+ 1581.71
1 b14 /1+ 1696.75
b16 /1+ 1719.81
1 b15 /1+ 1809.81
1 b16 /1+ 1922.89
Y0 /1+ 1923.91
138.05
145.05
163.06
168.06
186.08
204.09
325.11
366.14
Δm (ppm)
m/z

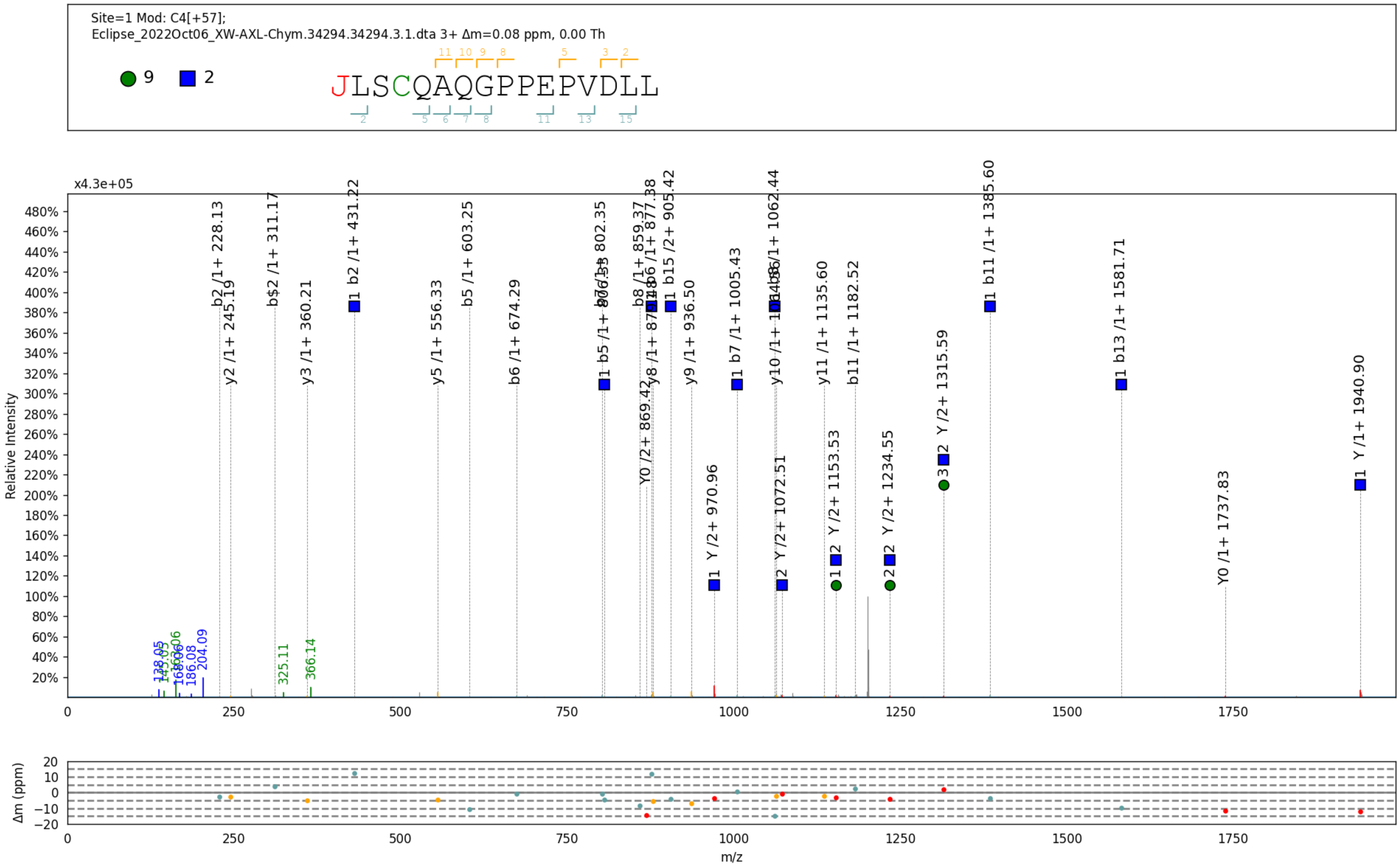

Site=1 Mod: C4[+57];
Eclipse_2022Oct06_XW-AXL-Chym.34294.34294.3.1.dta 3+ Δm=0.08 ppm, 0.00 Th
9
2
JLSCQAQGPPEPVDLL
x4.3e+05
Relative Intensity
m/z
Δm (ppm)

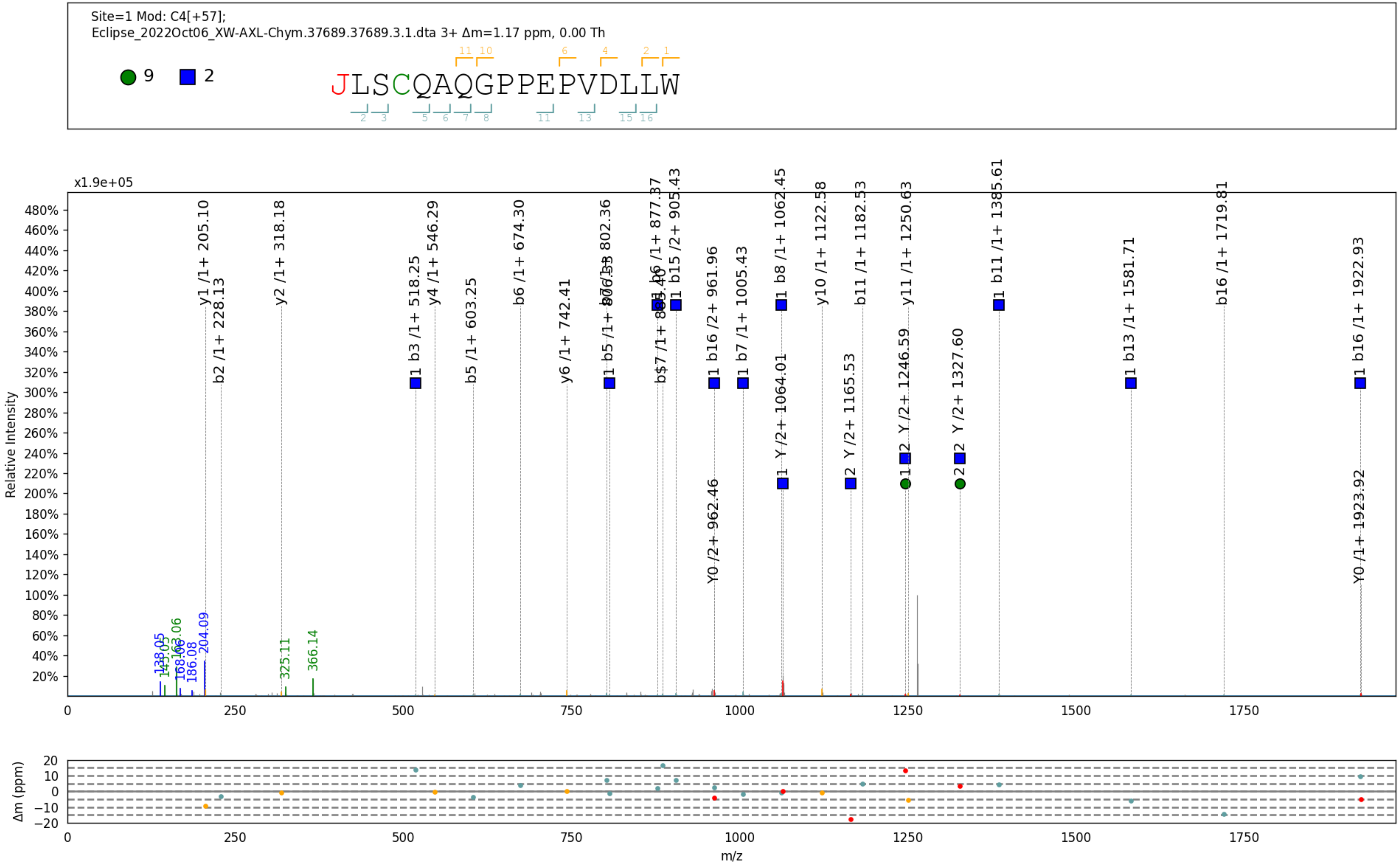
Site=1 Mod: C4[+57];
Eclipse_2022Oct06_XW-AXL-Chym.37689.37689.3.1.dta 3+ Δm=1.17 ppm, 0.00 Th
9
2
JLSCQAQGPPEPVDLLW
x1.9e+05
Relative Intensity
y1 /1+ 205.10
b2 /1+ 228.13
y2 /1+ 318.18
b3 /1+ 518.25
y4 /1+ 546.29
b5 /1+ 603.25
b6 /1+ 674.30
y6 /1+ 742.41
b7 /1+ 877.37
b15 /2+ 905.43
b16 /2+ 961.96
Y0 /2+ 962.46
b7 /1+ 1005.43
b8 /1+ 1062.45
Y /2+ 1064.01
y10 /1+ 1122.58
Y /2+ 1165.53
b11 /1+ 1182.53
Y /2+ 1246.59
y11 /1+ 1250.63
Y /2+ 1327.60
b11 /1+ 1385.61
b13 /1+ 1581.71
b16 /1+ 1719.81
b16 /1+ 1922.93
Y0 /1+ 1923.92
138.05
204.09
366.14
325.11
186.08
m/z
Δm (ppm)

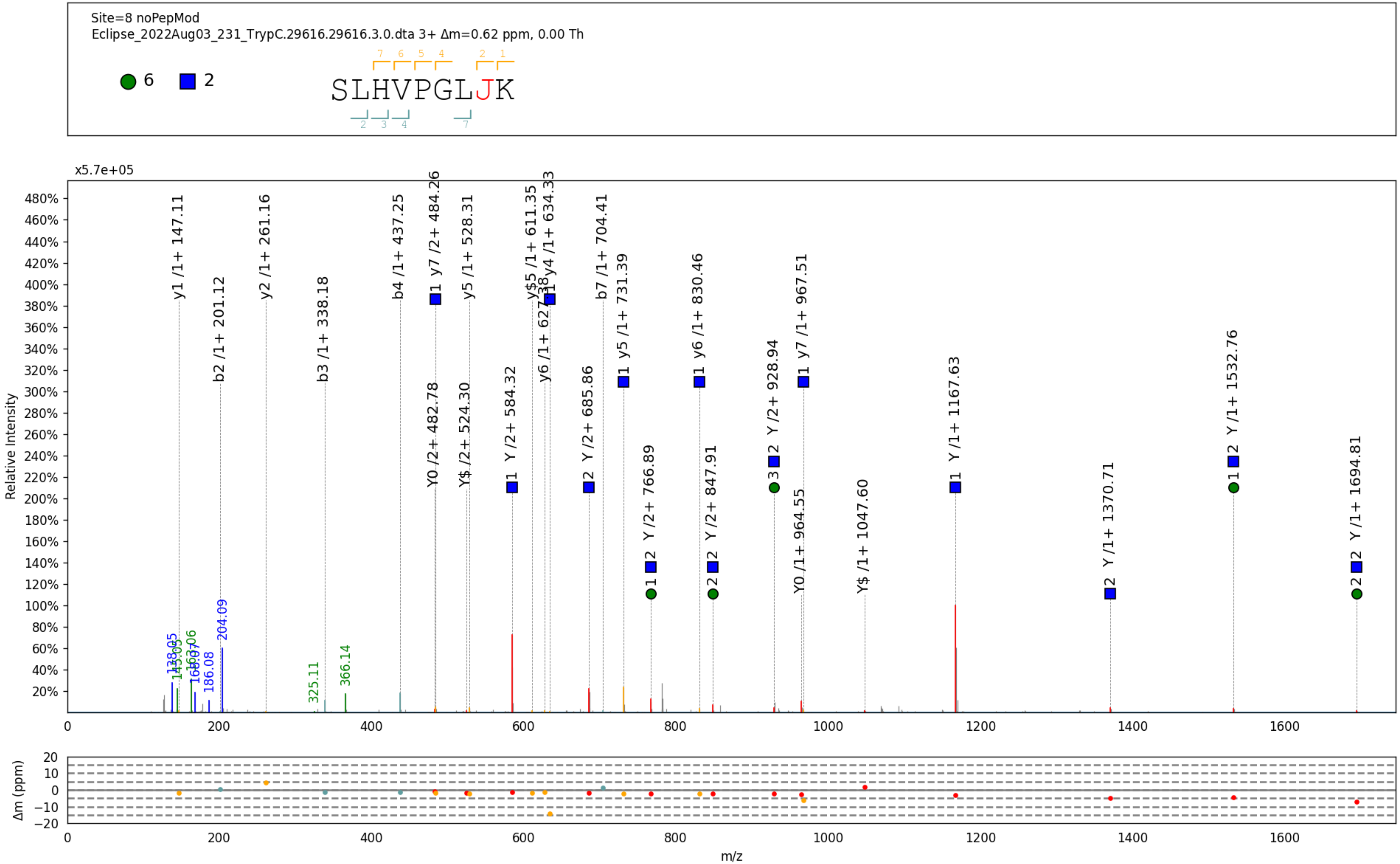

Site=8 noPepMod
Eclipse_2022Aug03_231_TrypC.29616.29616.3.0.dta 3+ Δm=0.62 ppm, 0.00 Th
6
2
SLHVPGLJK
x5.7e+05
Relative Intensity
m/z
Δm (ppm)
y1 /1+ 147.11
b2 /1+ 201.12
y2 /1+ 261.16
b3 /1+ 338.18
b4 /1+ 437.25
1 y7 /2+ 484.26
y5 /1+ 528.31
Y0 /2+ 482.78
Y$ /2+ 524.30
1 Y /2+ 584.32
y$5 /1+ 611.35
y6 /1+ 627.38
1 y4 /1+ 634.33
2 Y /2+ 685.86
b7 /1+ 704.41
1 y5 /1+ 731.39
1 2 Y /2+ 766.89
1 y6 /1+ 830.46
2 2 Y /2+ 847.91
3 2 Y /2+ 928.94
Y0 /1+ 964.55
1 y7 /1+ 967.51
Y$ /1+ 1047.60
1 Y /1+ 1167.63
2 Y /1+ 1370.71
1 2 Y /1+ 1532.76
2 2 Y /1+ 1694.81
138.05
143.09
163.06
168.07
186.08
204.09
325.11
366.14

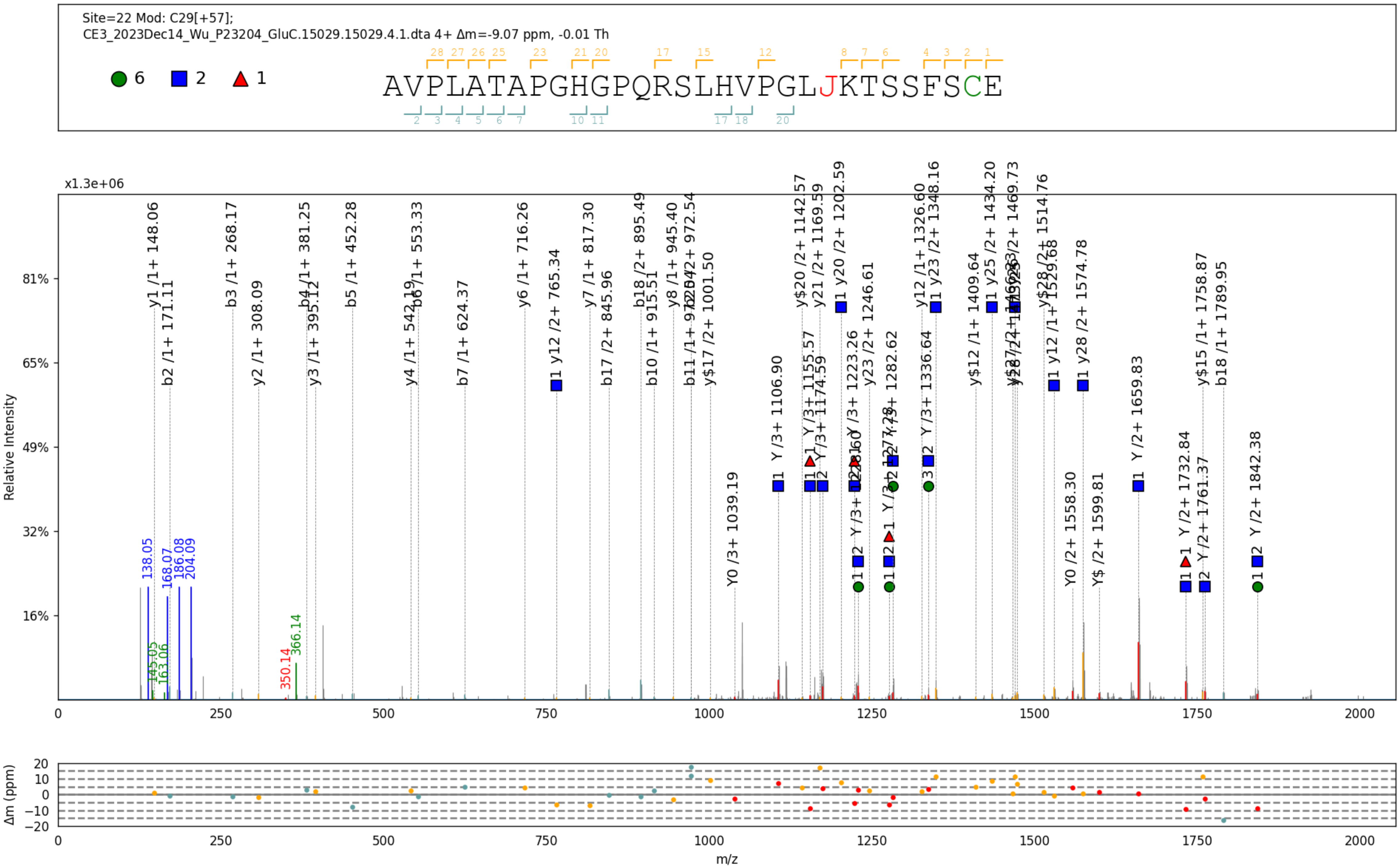
Site=22 Mod: C29[+57];
CE3_2023Dec14_Wu_P23204_GluC.15029.15029.4.1.dta 4+ Δm=-9.07 ppm, -0.01 Th
6
2
1
AVPLATAPGHGPQRSLHVPGLJKTSSFSCE
x1.3e+06
Relative Intensity
81%
65%
49%
32%
16%
y1 /1+ 148.06
b2 /1+ 171.11
b3 /1+ 268.17
y2 /1+ 308.09
b4 /1+ 381.25
y3 /1+ 395.12
b5 /1+ 452.28
y4 /1+ 542.19
b6 /1+ 553.33
b7 /1+ 624.37
y6 /1+ 716.26
1 y12 /2+ 765.34
y7 /1+ 817.30
b17 /2+ 845.96
b18 /2+ 895.49
b10 /1+ 915.51
y8 /1+ 945.40
y$17 /2+ 1001.50
Y0 /3+ 1039.19
1 Y /3+ 1106.90
y$20 /2+ 1142.57
y21 /2+ 1169.59
1 y20 /2+ 1202.59
y23 /2+ 1246.61
y12 /1+ 1326.60
1 y23 /2+ 1348.16
3 2 Y /3+ 1336.64
y$12 /1+ 1409.64
1 y25 /2+ 1434.20
y$28 /2+ 1514.76
1 y12 /1+ 1529.68
1 y28 /2+ 1574.78
Y0 /2+ 1558.30
Y$ /2+ 1599.81
1 Y /2+ 1659.83
1 1 Y /2+ 1732.84
y$15 /1+ 1758.87
2 Y /2+ 1761.37
b18 /1+ 1789.95
1 2 Y /2+ 1842.38
138.05
145.05
163.06
168.07
186.08
204.09
350.14
366.14
0
250
500
750
1000
1250
1500
1750
2000
Δm (ppm)
20
10
0
-10
-20
m/z

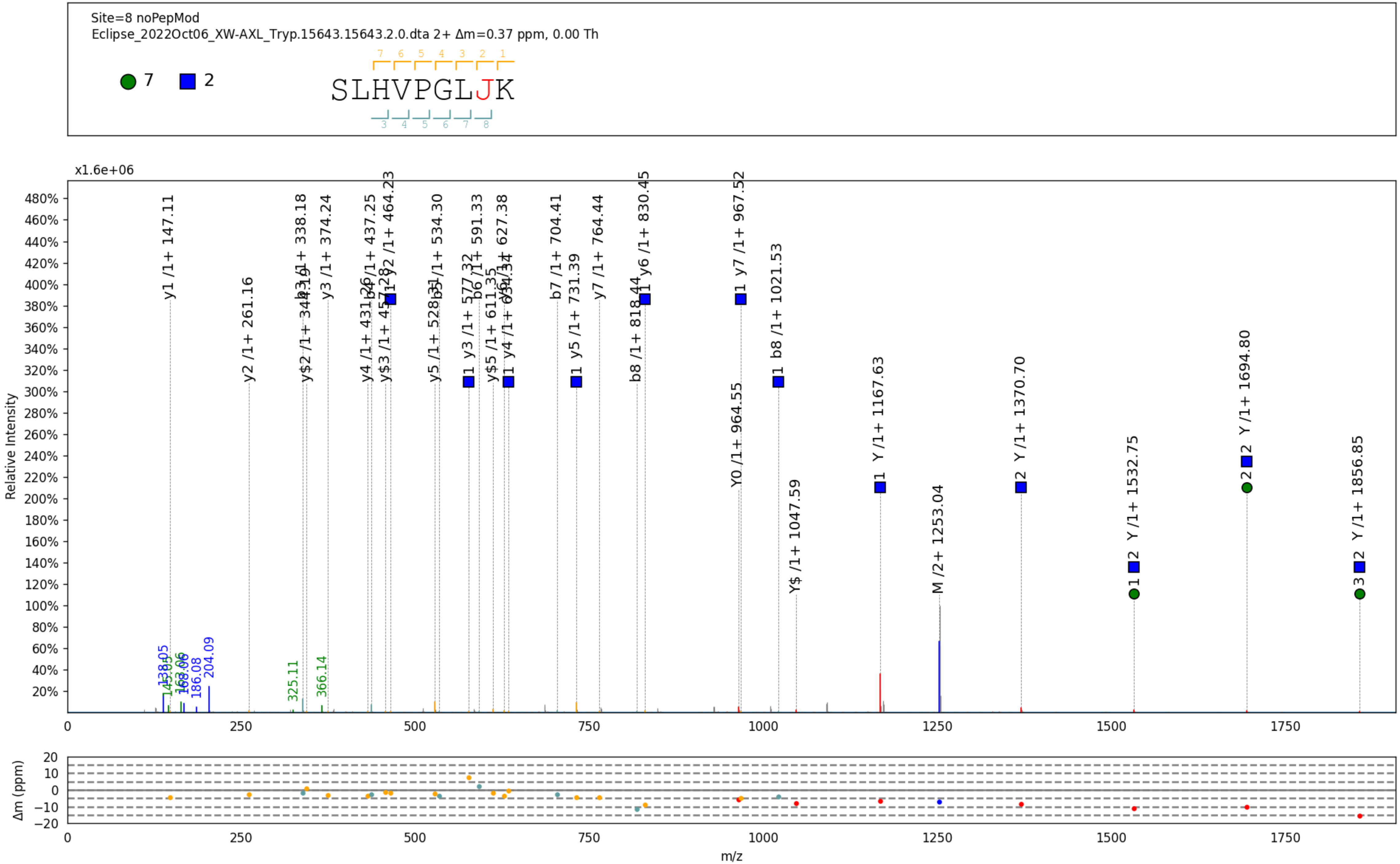
Site=8 noPepMod
Eclipse_2022Oct06_XW-AXL_Tryp.15643.15643.2.0.dta 2+ Δm=0.37 ppm, 0.00 Th
7
2
SLHVPGLJK
x1.6e+06
Relative Intensity
y1 /1+ 147.11
y2 /1+ 261.16
y3 /1+ 374.24
y4 /1+ 437.25
y2 /1+ 464.23
y5 /1+ 534.30
b6 /1+ 591.33
y4 /1+ 627.38
b7 /1+ 704.41
y5 /1+ 731.39
y7 /1+ 764.44
b8 /1+ 818.44
y6 /1+ 830.45
y7 /1+ 967.52
b8 /1+ 1021.53
Y0 /1+ 964.55
Y$ /1+ 1047.59
Y /1+ 1167.63
M /2+ 1253.04
Y /1+ 1370.70
Y /1+ 1532.75
Y /1+ 1694.80
Y /1+ 1856.85
138.05
163.06
186.08
204.09
325.11
366.14
Δm (ppm)
m/z

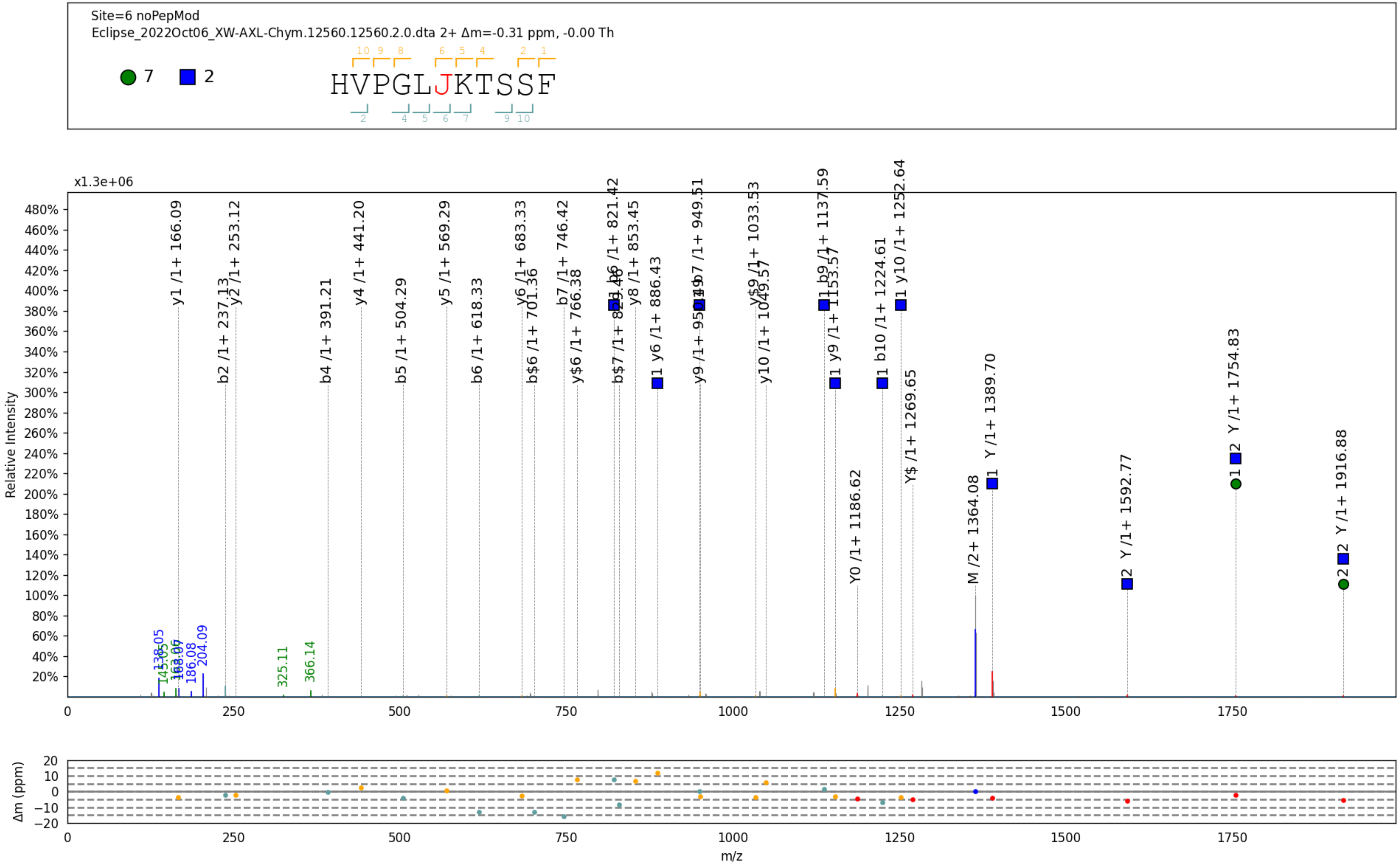

Site=6 noPepMod
Eclipse_2022Oct06_XW-AXL-Chym.12560.12560.2.0.dta 2+ Δm=-0.31 ppm, -0.00 Th
7
2
HVPGLJKTSSF
x1.3e+06
Relative Intensity
y1 /1+ 166.09
b2 /1+ 237.13
y2 /1+ 253.12
b4 /1+ 391.21
y4 /1+ 441.20
b5 /1+ 504.29
y5 /1+ 569.29
b6 /1+ 618.33
y6 /1+ 683.33
b$6 /1+ 701.36
b7 /1+ 746.42
y$6 /1+ 766.38
y8 /1+ 853.45
1 y6 /1+ 886.43
y9 /1+ 949.51
y10 /1+ 1049.57
1 y9 /1+ 1153.57
Y0 /1+ 1186.62
1 b10 /1+ 1224.61
1 y10 /1+ 1252.64
Y$ /1+ 1269.65
M /2+ 1364.08
1 Y /1+ 1389.70
2 Y /1+ 1592.77
1 2 Y /1+ 1754.83
2 2 Y /1+ 1916.88
138.05
145.05
163.07
168.06
186.08
204.09
325.11
366.14
m/z
Δm (ppm)

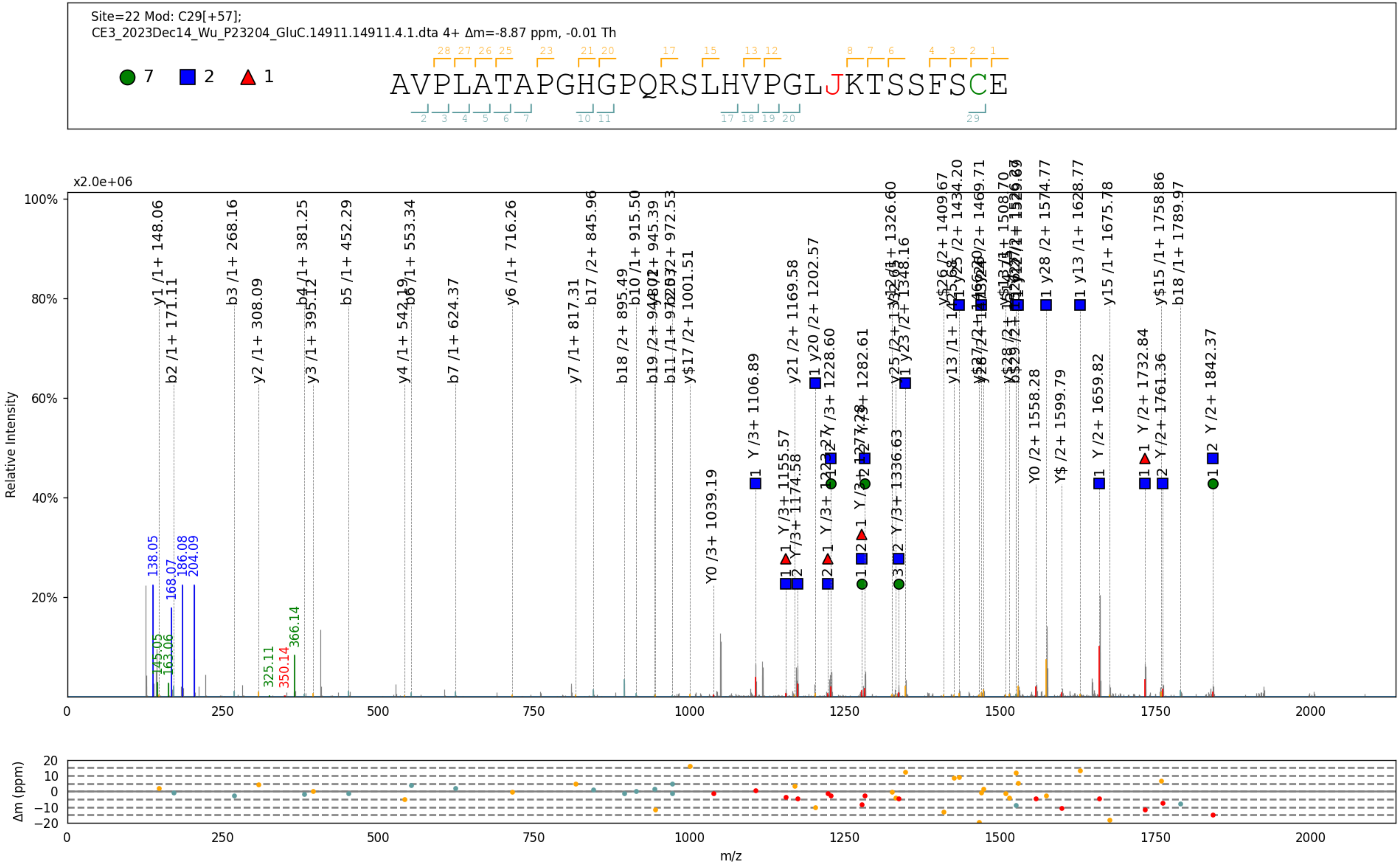
Site=22 Mod: C29[+57];
CE3_2023Dec14_Wu_P23204_GluC.14911.14911.4.1.dta 4+ Δm=-8.87 ppm, -0.01 Th
7
2
1
AVPLATAPGHGPQRSLHVPGLJKTSSFSCE
x2.0e+06
Relative Intensity
y1 /1+ 148.06
b2 /1+ 171.11
b3 /1+ 268.16
y2 /1+ 308.09
b4 /1+ 381.25
y3 /1+ 395.12
b5 /1+ 452.29
y4 /1+ 542.19
b6 /1+ 553.34
b7 /1+ 624.37
y6 /1+ 716.26
y7 /1+ 817.31
b17 /2+ 845.96
b18 /2+ 895.49
b10 /1+ 915.50
y$17 /2+ 1001.51
Y0 /3+ 1039.19
Y /3+ 1106.89
Y /3+ 1155.57
Y /3+ 1174.58
y21 /2+ 1169.58
y20 /2+ 1202.57
Y /3+ 1228.60
Y /3+ 1282.61
y25 /2+ 1326.60
Y /3+ 1336.63
y23 /2+ 1348.16
y$26 /2+ 1409.67
y25 /2+ 1434.20
1469.71
y28 /2+ 1574.77
Y0 /2+ 1558.28
Y$ /2+ 1599.79
y13 /1+ 1628.77
Y /2+ 1659.82
y15 /1+ 1675.78
Y /2+ 1732.84
Y /2+ 1761.36
y$15 /1+ 1758.86
b18 /1+ 1789.97
Y /2+ 1842.37
138.05
168.07
186.08
204.09
145.05
163.06
325.11
350.14
366.14
Δm (ppm)
m/z

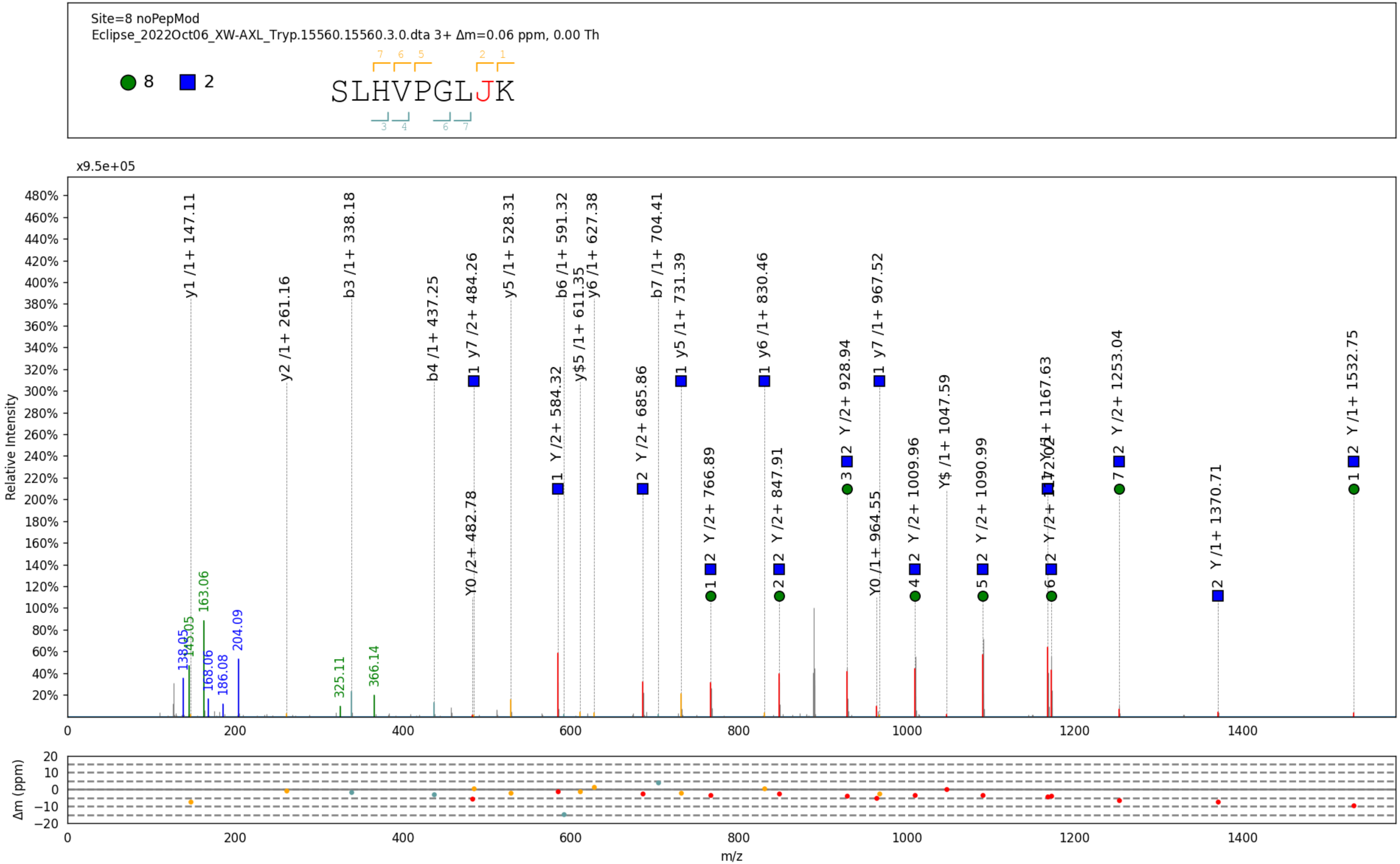

Site=8 noPepMod
Eclipse_2022Oct06_XW-AXL_Tryp.15560.15560.3.0.dta 3+ Δm=0.06 ppm, 0.00 Th
8
2
SLHVPGLJK
x9.5e+05
Relative Intensity
m/z
Δm (ppm)
y1 /1+ 147.11
y2 /1+ 261.16
b3 /1+ 338.18
b4 /1+ 437.25
1 y7 /2+ 484.26
Y0 /2+ 482.78
y5 /1+ 528.31
1 Y /2+ 584.32
b6 /1+ 591.32
y$5 /1+ 611.35
y6 /1+ 627.38
2 Y /2+ 685.86
b7 /1+ 704.41
1 y5 /1+ 731.39
1 2 Y /2+ 766.89
1 y6 /1+ 830.46
2 2 Y /2+ 847.91
3 2 Y /2+ 928.94
Y0 /1+ 964.55
1 y7 /1+ 967.52
4 2 Y /2+ 1009.96
Y$ /1+ 1047.59
5 2 Y /2+ 1090.99
1 Y /1+ 1167.63
6 2 Y /2+ 1172.02
7 2 Y /2+ 1253.04
2 Y /1+ 1370.71
1 2 Y /1+ 1532.75
138.05
145.05
163.06
168.06
186.08
204.09
325.11
366.14

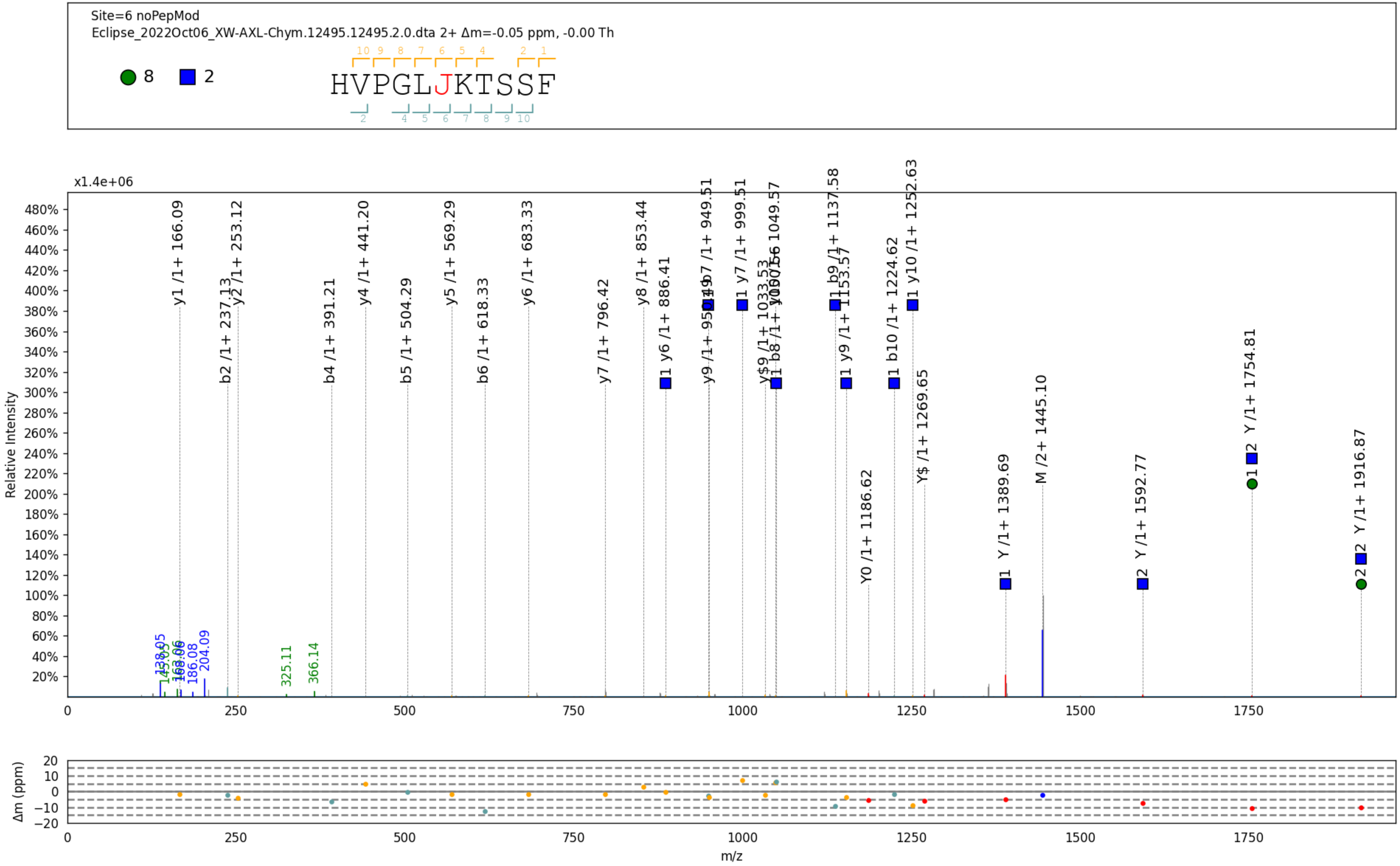

Site=6 noPepMod
Eclipse_2022Oct06_XW-AXL-Chym.12495.12495.2.0.dta 2+ Δm=-0.05 ppm, -0.00 Th
8
2
HVPGLJKTSSF
x1.4e+06
Relative Intensity
y1 /1+ 166.09
b2 /1+ 237.13
y2 /1+ 253.12
b4 /1+ 391.21
y4 /1+ 441.20
b5 /1+ 504.29
y5 /1+ 569.29
b6 /1+ 618.33
y6 /1+ 683.33
y7 /1+ 796.42
y8 /1+ 853.44
1 y6 /1+ 886.41
y9 /1+ 950.49
1 y7 /1+ 999.51
y$9 /1+ 1033.53
1 b8 /1+ 1049.57
1 b9 /1+ 1137.58
1 y9 /1+ 1153.57
Y0 /1+ 1186.62
1 b10 /1+ 1224.62
1 y10 /1+ 1252.63
Y$ /1+ 1269.65
1 Y /1+ 1389.69
M /2+ 1445.10
2 Y /1+ 1592.77
1 2 Y /1+ 1754.81
2 2 Y /1+ 1916.87
138.05
143.05
163.06
168.06
186.08
204.09
325.11
366.14
m/z
Δm (ppm)

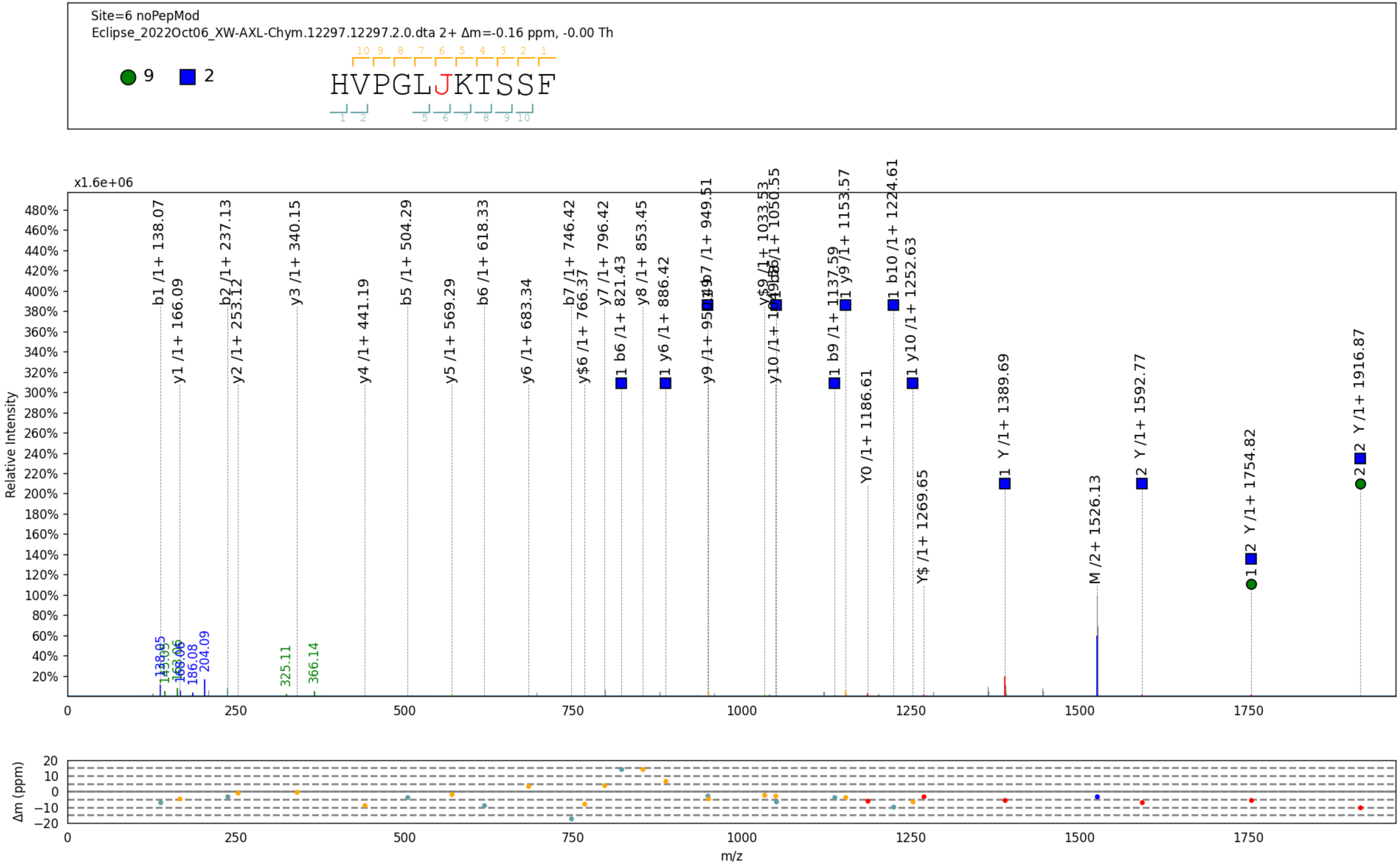
Site=6 noPepMod
Eclipse_2022Oct06_XW-AXL-Chym.12297.12297.2.0.dta 2+ Δm=-0.16 ppm, -0.00 Th
9
2
HVPGLJKTSSF
x1.6e+06
Relative Intensity
b1 /1+ 138.07
y1 /1+ 166.09
b2 /1+ 237.13
y2 /1+ 253.12
y3 /1+ 340.15
y4 /1+ 441.19
b5 /1+ 504.29
y5 /1+ 569.29
b6 /1+ 618.33
y6 /1+ 683.34
b7 /1+ 746.42
y$6 /1+ 766.37
y7 /1+ 796.42
1 b6 /1+ 821.43
y8 /1+ 853.45
1 y6 /1+ 886.42
y9 /1+ 949.51
y$9 /1+ 1033.53
y10 /1+ 1050.55
1 b9 /1+ 1137.59
1 y9 /1+ 1153.57
Y0 /1+ 1186.61
1 b10 /1+ 1224.61
1 y10 /1+ 1252.63
Y$ /1+ 1269.65
1 Y /1+ 1389.69
M /2+ 1526.13
2 Y /1+ 1592.77
1 2 Y /1+ 1754.82
2 2 Y /1+ 1916.87
138.05
145.09
163.06
168.06
186.08
204.09
325.11
366.14
Δm (ppm)
m/z

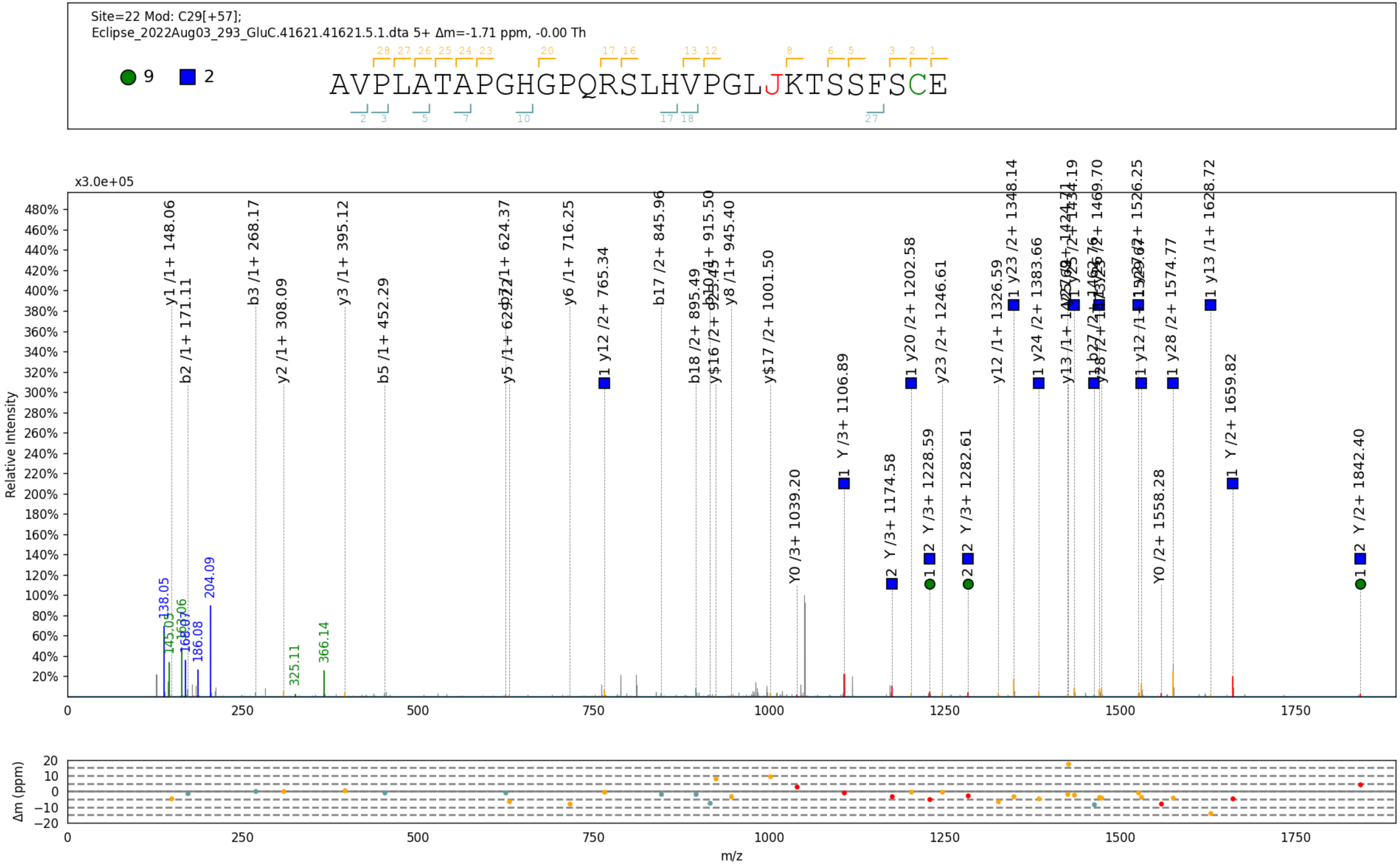

Site=22 Mod: C29[+57];
Eclipse_2022Aug03_293_GluC.41621.41621.5.1.dta 5+ Δm=-1.71 ppm, -0.00 Th
9
2
AVPLATAPGHGPQRSLHVPGLJKTSSFSCE
x3.0e+05
Relative Intensity
y1 /1+ 148.06
b2 /1+ 171.11
b3 /1+ 268.17
y2 /1+ 308.09
y3 /1+ 395.12
b5 /1+ 452.29
y6 /1+ 716.25
1 y12 /2+ 765.34
b17 /2+ 845.96
b18 /2+ 895.49
y8 /1+ 945.40
y$17 /2+ 1001.50
Y0 /3+ 1039.20
1 Y /3+ 1106.89
2 Y /3+ 1174.58
1 y20 /2+ 1202.58
2 Y /3+ 1228.59
y23 /2+ 1246.61
2 Y /3+ 1282.61
y12 /1+ 1326.59
1 y23 /2+ 1348.14
1 y24 /2+ 1383.66
Y0 /2+ 1558.28
1 y28 /2+ 1574.77
1 y13 /1+ 1628.72
1 Y /2+ 1659.82
2 Y /2+ 1842.40
138.05
204.09
186.08
325.11
366.14
m/z
Δm (ppm)

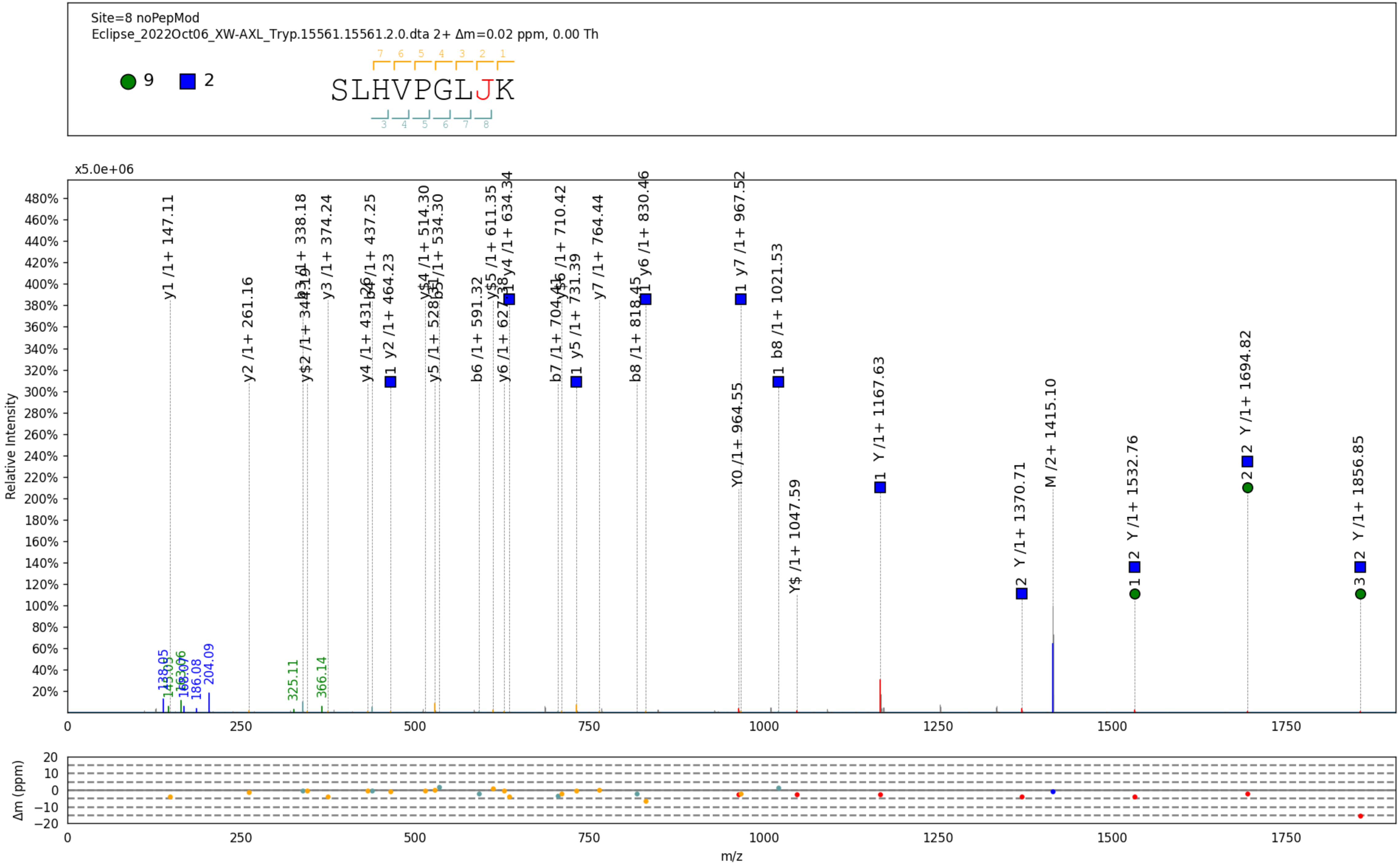

Site=8 noPepMod
Eclipse_2022Oct06_XW-AXL_Tryp.15561.15561.2.0.dta 2+ Δm=0.02 ppm, 0.00 Th
9
2
SLHVPGLJK
x5.0e+06
Relative Intensity
y1 /1+ 147.11
y2 /1+ 261.16
b3 /1+ 338.18
y3 /1+ 374.24
b4 /1+ 437.25
y4 /1+ 431.26
1 y2 /1+ 464.23
y5 /1+ 528.31
b5 /1+ 534.30
y$4 /1+ 514.30
b6 /1+ 591.32
y$5 /1+ 611.35
y6 /1+ 627.38
1 y4 /1+ 634.34
b7 /1+ 704.41
y$6 /1+ 710.42
1 y5 /1+ 731.39
y7 /1+ 764.44
b8 /1+ 818.45
1 y6 /1+ 830.46
Y0 /1+ 964.55
1 y7 /1+ 967.52
1 b8 /1+ 1021.53
Y$ /1+ 1047.59
1 Y /1+ 1167.63
2 Y /1+ 1370.71
M /2+ 1415.10
1 2 Y /1+ 1532.76
2 2 Y /1+ 1694.82
3 2 Y /1+ 1856.85
138.05
163.06
186.08
204.09
325.11
366.14
Δm (ppm)
m/z

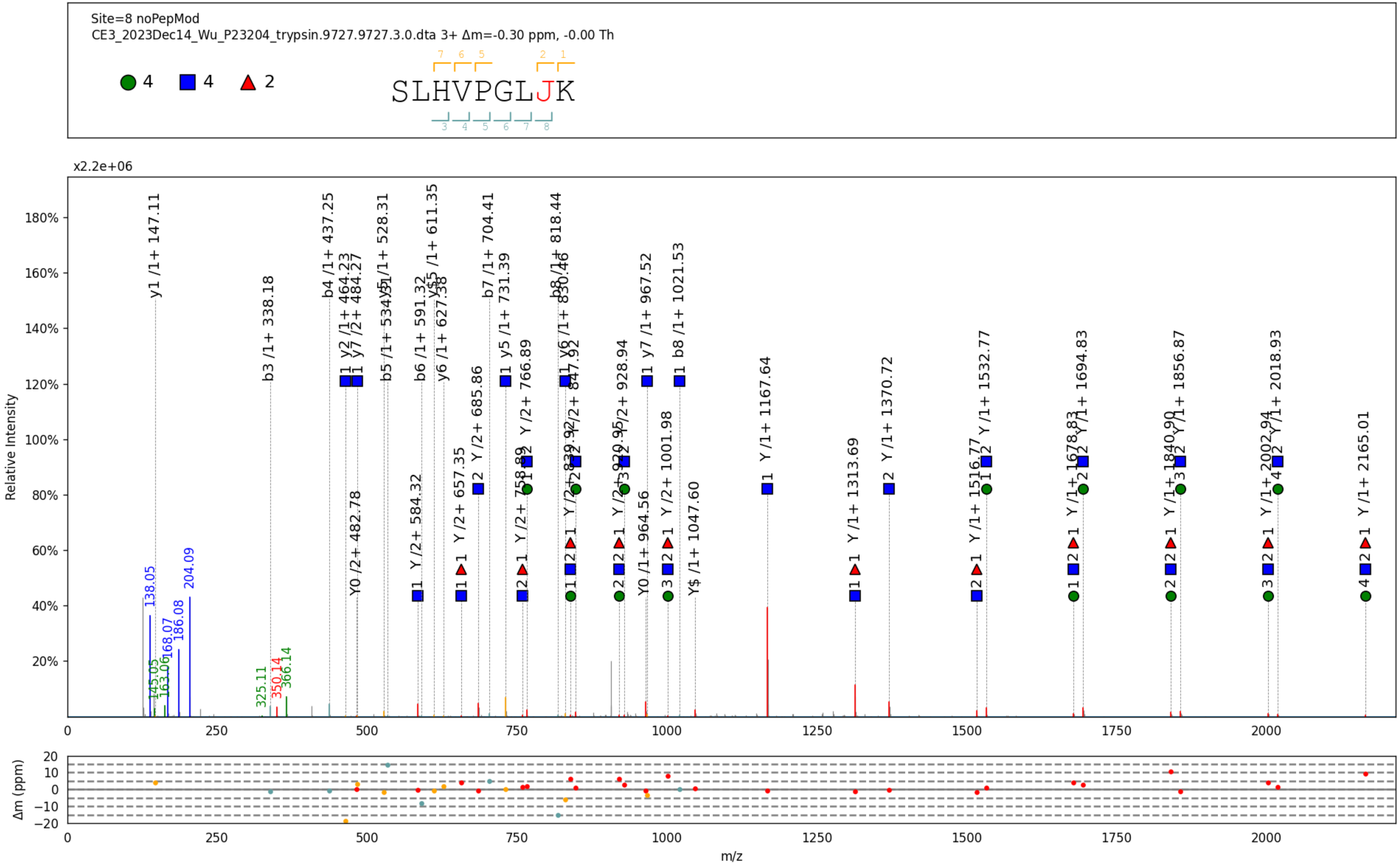
Site=8 noPepMod
CE3_2023Dec14_Wu_P23204_trypsin.9727.9727.3.0.dta 3+ Δm=-0.30 ppm, -0.00 Th
4 4 2
SLHVPGLJK
x2.2e+06
Relative Intensity
m/z
Δm (ppm)
y1 /1+ 147.11
b3 /1+ 338.18
b4 /1+ 437.25
y2 /1+ 464.23
y7 /2+ 484.27
Y0 /2+ 482.78
b5 /1+ 528.31
b6 /1+ 591.32
y6 /1+ 627.38
Y$ /1+ 611.35
b7 /1+ 704.41
y5 /1+ 731.39
b8 /1+ 818.44
y6 /1+ 830.46
y7 /1+ 967.52
b8 /1+ 1021.53
Y0 /1+ 964.56
Y$ /1+ 1047.60
Y /1+ 1167.64
Y /1+ 1313.69
Y /1+ 1370.72
Y /1+ 1516.77
Y /1+ 1532.77
Y /1+ 1678.83
Y /1+ 1694.83
Y /1+ 1840.90
Y /1+ 1856.87
Y /1+ 2002.94
Y /1+ 2018.93
Y /1+ 2165.01
138.05
145.05
163.06
168.07
186.08
204.09
325.11
350.14
366.14

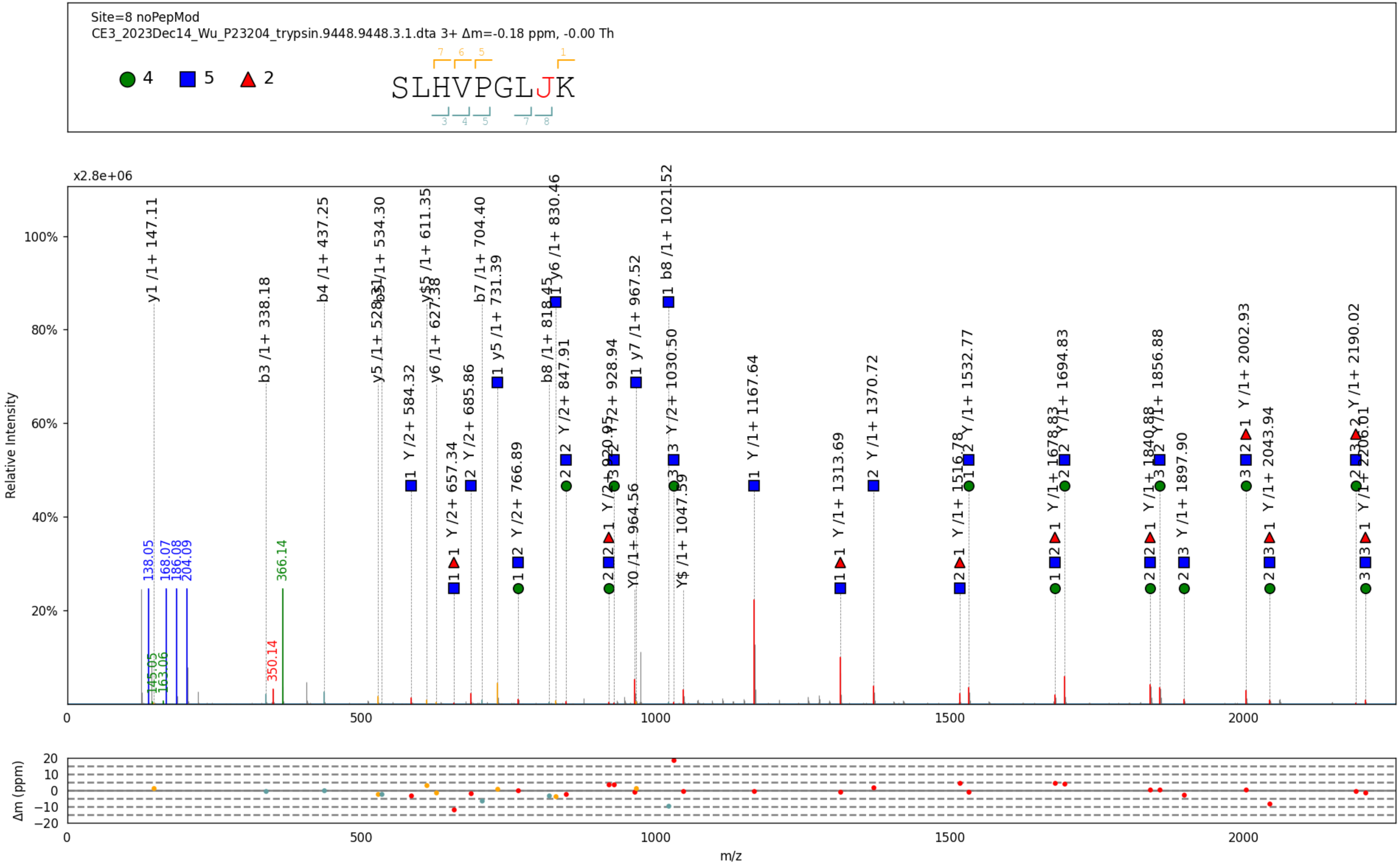

Site=8 noPepMod
CE3_2023Dec14_Wu_P23204_trypsin.9448.9448.3.1.dta 3+ Δm=-0.18 ppm, -0.00 Th
4
5
2
SLHVPGLJK
x2.8e+06
Relative Intensity
m/z
Δm (ppm)
y1 /1+ 147.11
b3 /1+ 338.18
b4 /1+ 437.25
y5 /1+ 528.31
b5 /1+ 534.30
y$5 /1+ 611.35
y6 /1+ 627.38
b7 /1+ 704.40
1 y5 /1+ 731.39
b8 /1+ 818.45
1 y6 /1+ 830.46
1 y7 /1+ 967.52
Y0 /1+ 964.56
1 b8 /1+ 1021.52
Y$ /1+ 1047.59
138.05
168.07
186.08
204.09
366.14
350.14
145.05
163.06

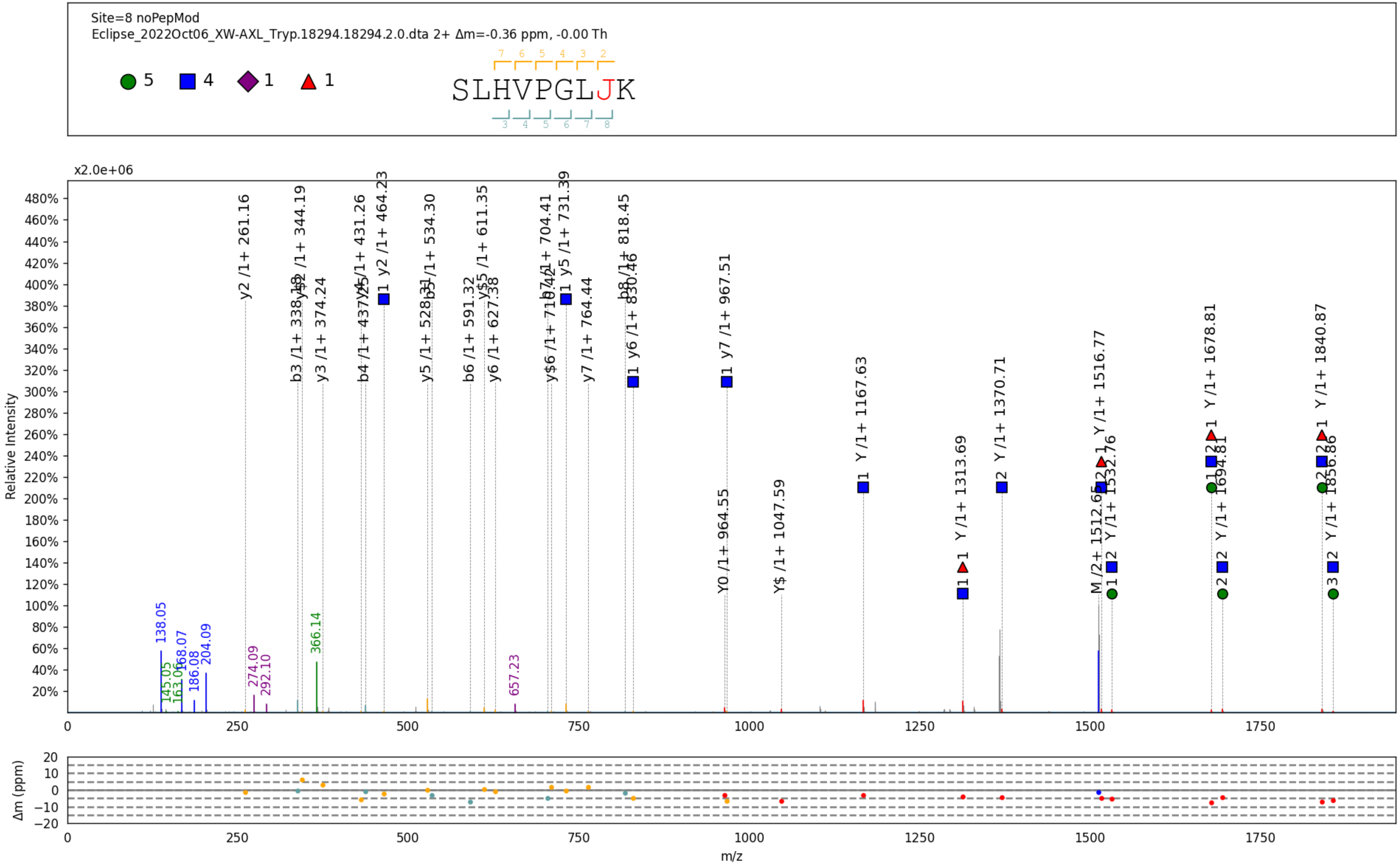
Site=8 noPepMod
Eclipse_2022Oct06_XW-AXL_Tryp.18294.18294.2.0.dta 2+ Δm=-0.36 ppm, -0.00 Th
5 4 1 1
SLHVPGLJK
x2.0e+06
Relative Intensity
m/z
Δm (ppm)
y2 /1+ 261.16
b3 /1+ 338.19
y$2 /1+ 344.19
y3 /1+ 374.24
b4 /1+ 431.26
y$3 /1+ 437.25
1 y2 /1+ 464.23
y5 /1+ 528.31
b5 /1+ 534.30
b6 /1+ 591.32
y$5 /1+ 611.35
y6 /1+ 627.38
b7 /1+ 704.41
y$6 /1+ 710.42
1 y5 /1+ 731.39
y7 /1+ 764.44
b8 /1+ 818.45
1 y6 /1+ 830.46
1 y7 /1+ 967.51
Y0 /1+ 964.55
Y$ /1+ 1047.59
1 Y /1+ 1167.63
1 1 Y /1+ 1313.69
2 Y /1+ 1370.71
M /2+ 1512.65
1 2 1 Y /1+ 1516.77
1 2 Y /1+ 1532.76
1 2 1 Y /1+ 1678.81
2 2 Y /1+ 1694.81
1 2 2 1 Y /1+ 1840.87
3 2 Y /1+ 1856.86
138.05
145.05
163.06
168.07
186.08
204.09
274.09
292.10
366.14
657.23

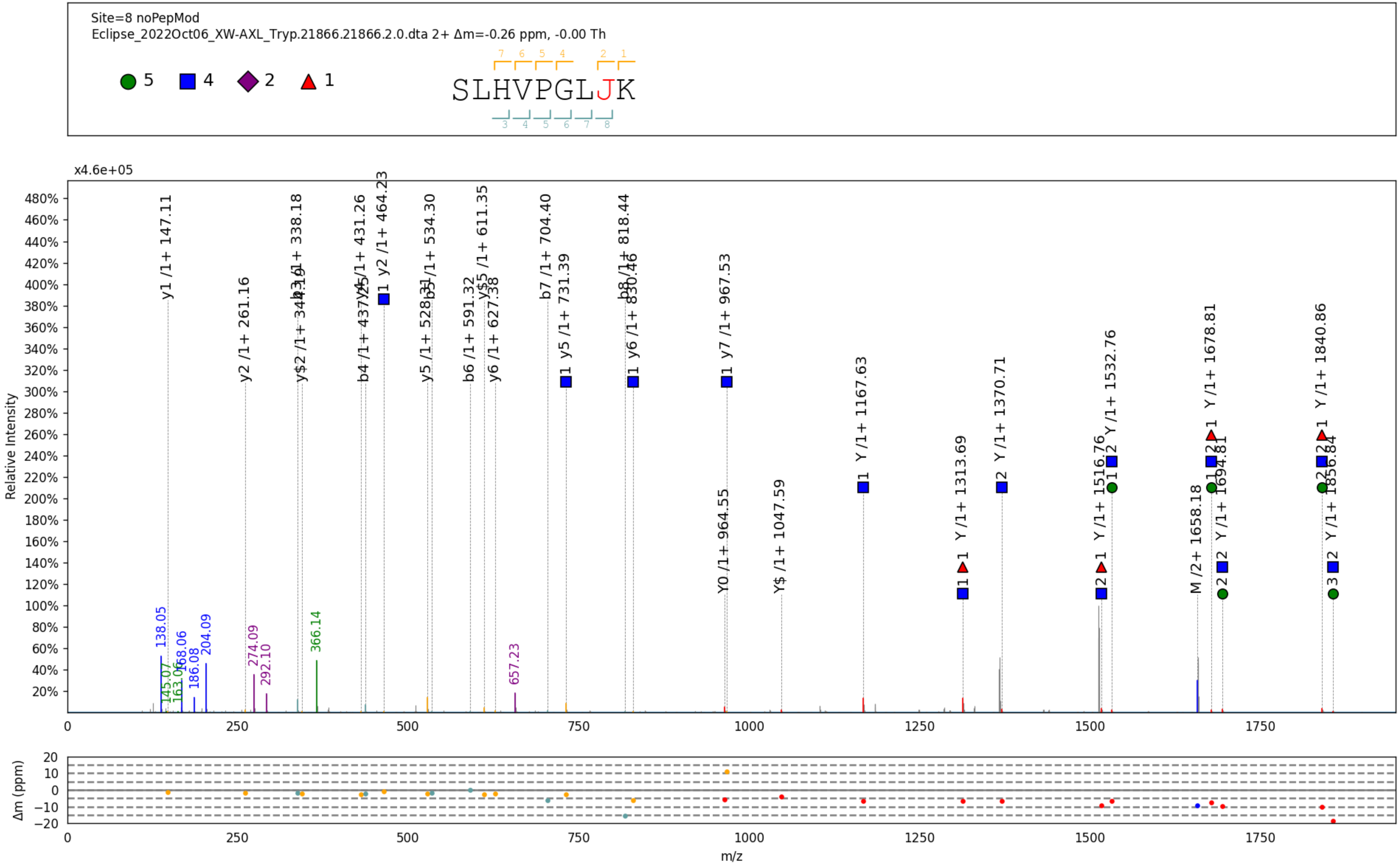

Site=8 noPepMod
Eclipse_2022Oct06_XW-AXL_Tryp.21866.21866.2.0.dta 2+ Δm=-0.26 ppm, -0.00 Th
5
4
2
1
SLHVPGLJK
x4.6e+05
Relative Intensity
y1 /1+ 147.11
y2 /1+ 261.16
b3 /1+ 338.18
y$2 /1+ 344.19
b4 /1+ 431.26
y$5 /1+ 437.25
1 y2 /1+ 464.23
y5 /1+ 528.31
b5 /1+ 534.30
b6 /1+ 591.32
y$5 /1+ 611.35
y6 /1+ 627.38
b7 /1+ 704.40
1 y5 /1+ 731.39
b8 /1+ 818.44
1 y6 /1+ 830.46
1 y7 /1+ 967.53
Y0 /1+ 964.55
Y$ /1+ 1047.59
1 Y /1+ 1167.63
1 1 Y /1+ 1313.69
2 Y /1+ 1370.71
2 1 Y /1+ 1516.76
1 2 Y /1+ 1532.76
M /2+ 1658.18
1 2 1 Y /1+ 1678.81
2 2 Y /1+ 1694.81
2 2 1 Y /1+ 1840.86
3 2 Y /1+ 1856.84
138.05
145.07
163.06
168.06
186.08
204.09
274.09
292.10
366.14
657.23
Δm (ppm)
m/z

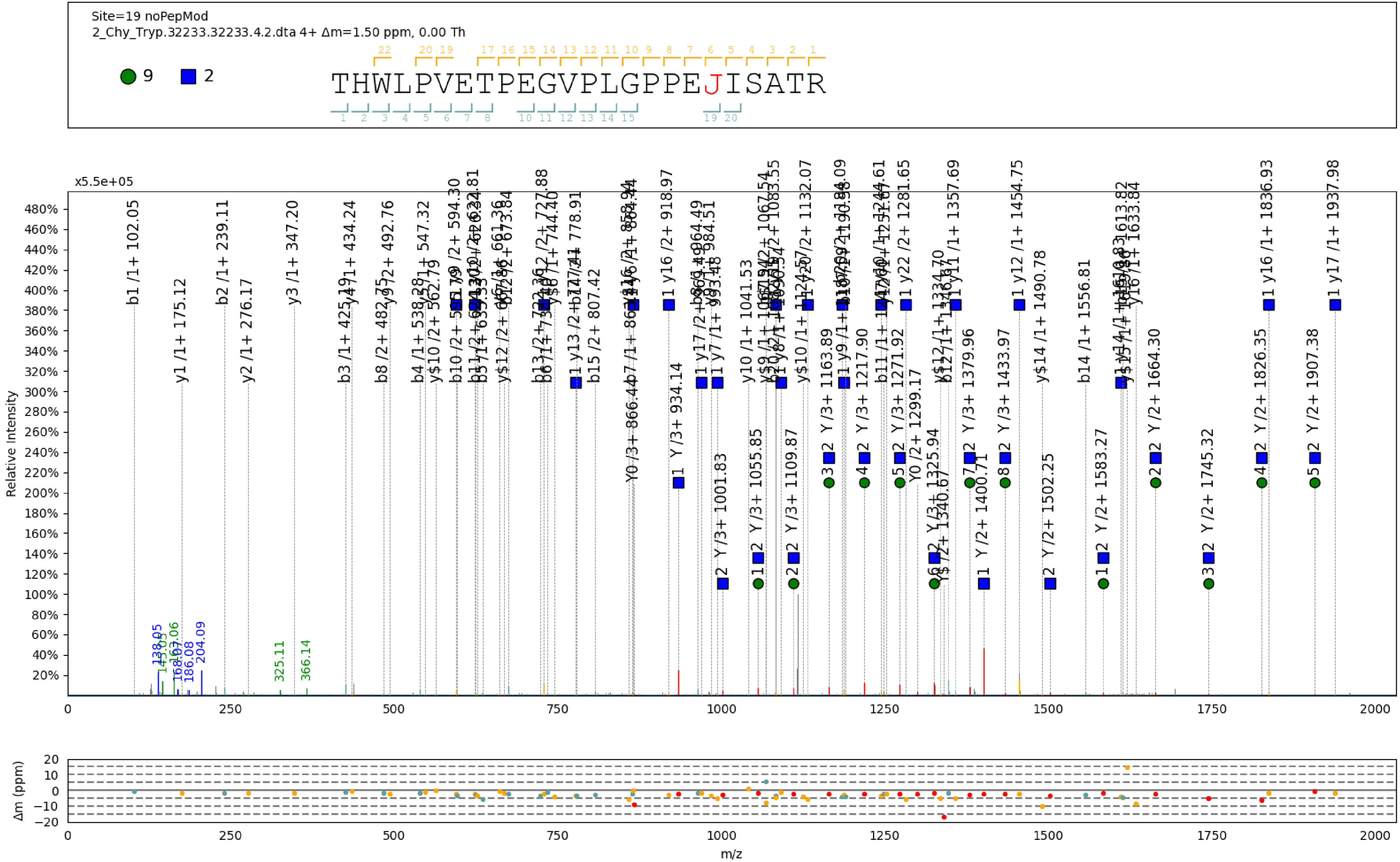

Site=19 noPepMod
2_Chy_Tryp.32233.32233.4.2.dta 4+ Δm=1.50 ppm, 0.00 Th
9
2
THWLPVETPEGVPLGPPEJISATR
x5.5e+05
Relative Intensity
m/z
Δm (ppm)

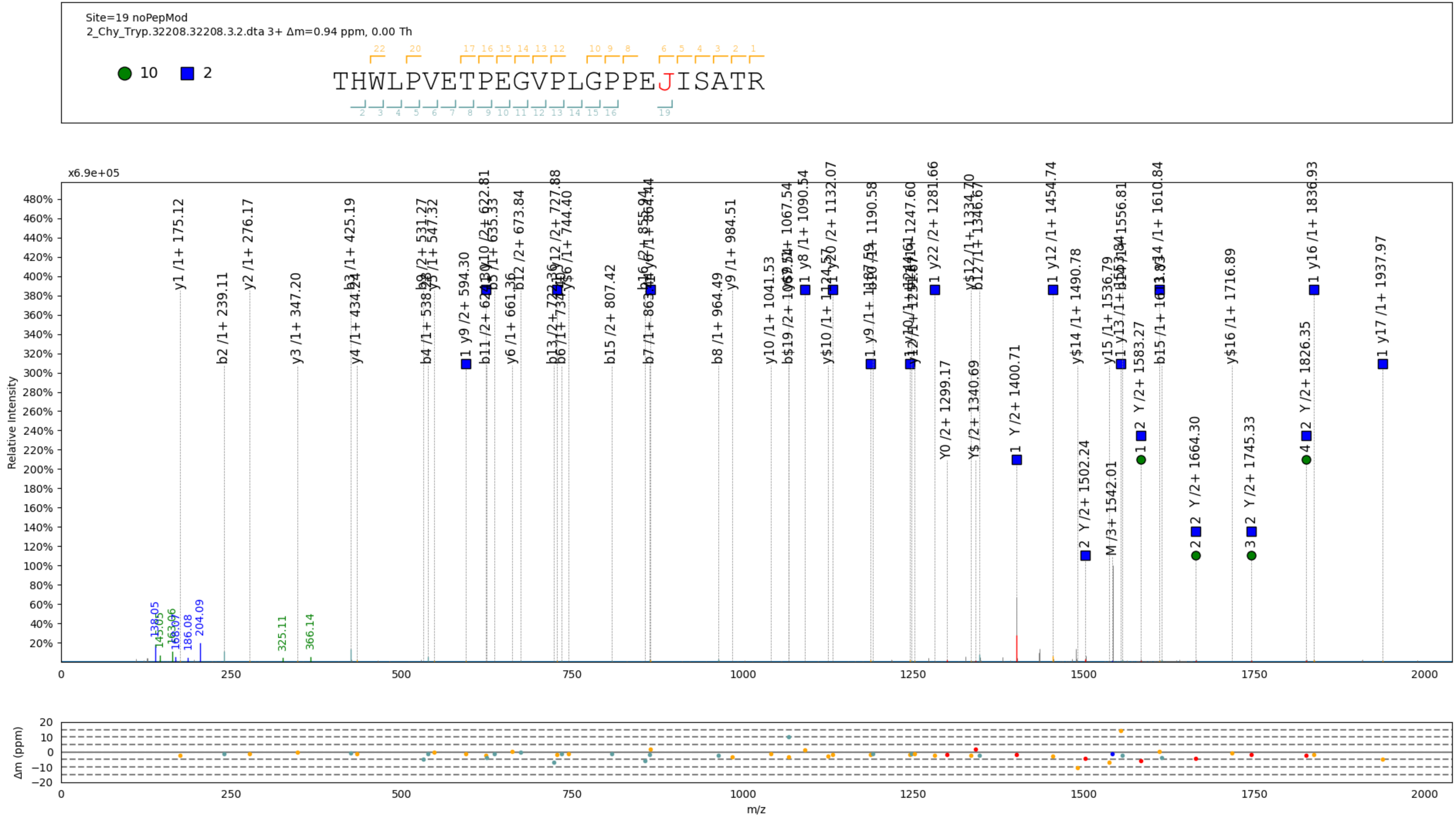

Site=19 noPepMod
2_Chy_Tryp.32208.32208.3.2.dta 3+ Δm=0.94 ppm, 0.00 Th
10
2
THWLPVETPEGVPLGPPEJISATR
x6.9e+05
Relative Intensity
m/z
Δm (ppm)

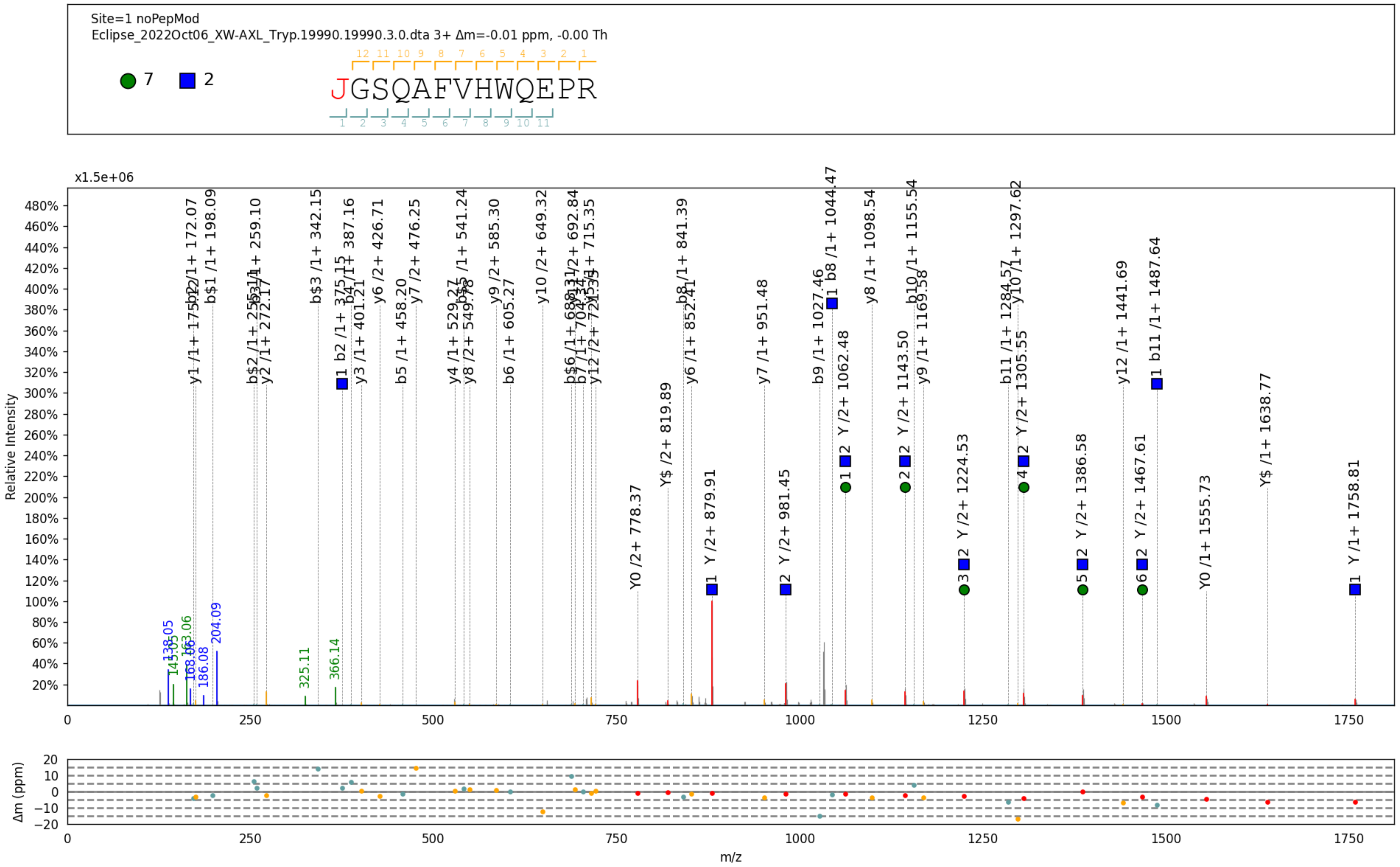
Site=1 noPepMod
Eclipse_2022Oct06_XW-AXL_Tryp.19990.19990.3.0.dta 3+ Δm=-0.01 ppm, -0.00 Th
7
2
JGSQAFVHWQEPR
x1.5e+06
Relative Intensity
y1 /1+ 175.12
b2 /1+ 172.07
b$1 /1+ 198.09
b$2 /1+ 255.11
b3 /1+ 259.10
y2 /1+ 272.17
b$3 /1+ 342.15
1 b2 /1+ 375.15
b4 /1+ 387.16
y3 /1+ 401.21
y6 /2+ 426.71
b5 /1+ 458.20
y7 /2+ 476.25
y4 /1+ 529.27
b$5 /1+ 541.24
y8 /2+ 549.78
y9 /2+ 585.30
b6 /1+ 605.27
y10 /2+ 649.32
Y0 /2+ 778.37
Y$ /2+ 819.89
b8 /1+ 841.39
y6 /1+ 852.41
1 Y /2+ 879.91
y7 /1+ 951.48
2 Y /2+ 981.45
b9 /1+ 1027.46
1 b8 /1+ 1044.47
1 2 Y /2+ 1062.48
y8 /1+ 1098.54
2 2 Y /2+ 1143.50
b10 /1+ 1155.54
y9 /1+ 1169.58
3 2 Y /2+ 1224.53
b11 /1+ 1284.57
y10 /1+ 1297.62
4 2 Y /2+ 1305.55
5 2 Y /2+ 1386.58
y12 /1+ 1441.69
6 2 Y /2+ 1467.61
1 b11 /1+ 1487.64
Y0 /1+ 1555.73
Y$ /1+ 1638.77
1 Y /1+ 1758.81
138.05
145.05
163.06
168.06
186.08
204.09
325.11
366.14
m/z
Δm (ppm)

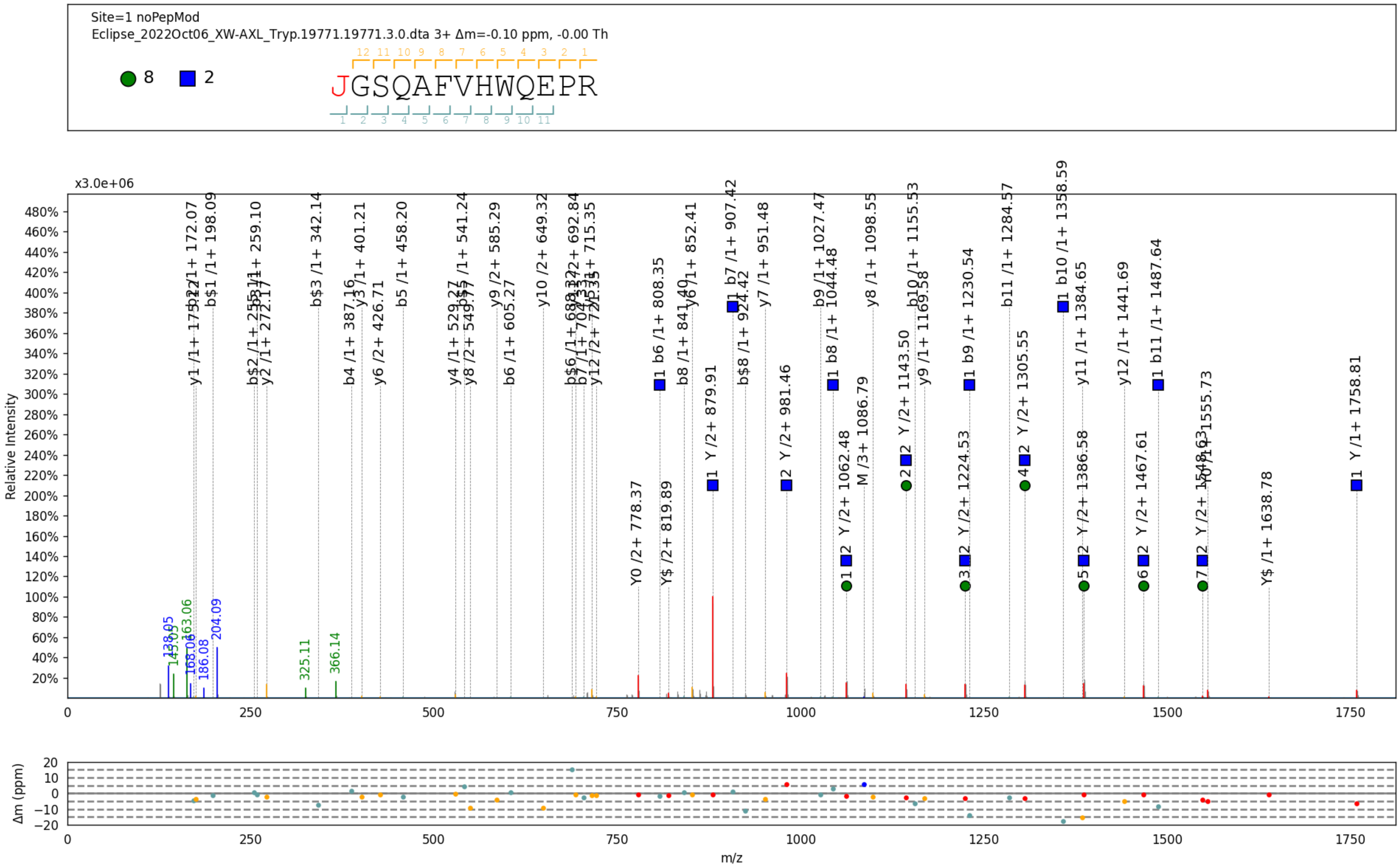

Site=1 noPepMod
Eclipse_2022Oct06_XW-AXL_Tryp.19771.19771.3.0.dta 3+ Δm=-0.10 ppm, -0.00 Th
8
2
JGSQAFVHWQEPR
x3.0e+06
Relative Intensity
y1 /1+ 175.12
b$1 /1+ 198.09
y2 /1+ 272.17
b$3 /1+ 342.14
b4 /1+ 387.16
y3 /1+ 401.21
y6 /2+ 426.71
b5 /1+ 458.20
y4 /1+ 529.27
y8 /2+ 549.77
y9 /2+ 585.29
b6 /1+ 605.27
y10 /2+ 649.32
YO /2+ 778.37
b6 /1+ 808.35
Y$ /2+ 819.89
b8 /1+ 841.40
y6 /1+ 852.41
Y /2+ 879.91
b7 /1+ 907.42
b$8 /1+ 924.42
y7 /1+ 951.48
Y /2+ 981.46
b9 /1+ 1027.47
b8 /1+ 1044.48
Y /2+ 1062.48
M /3+ 1086.79
y8 /1+ 1098.55
Y /2+ 1143.50
b10 /1+ 1155.53
y9 /1+ 1169.58
Y /2+ 1224.53
b9 /1+ 1230.54
b11 /1+ 1284.57
Y /2+ 1305.55
b10 /1+ 1358.59
y11 /1+ 1384.65
Y /2+ 1386.58
y12 /1+ 1441.69
Y /2+ 1467.61
b11 /1+ 1487.64
Y /2+ 1548.63
Y0 /1+ 1555.73
Y$ /1+ 1638.78
Y /1+ 1758.81
138.05
145.05
163.06
168.06
186.08
204.09
325.11
366.14
m/z
Δm (ppm)

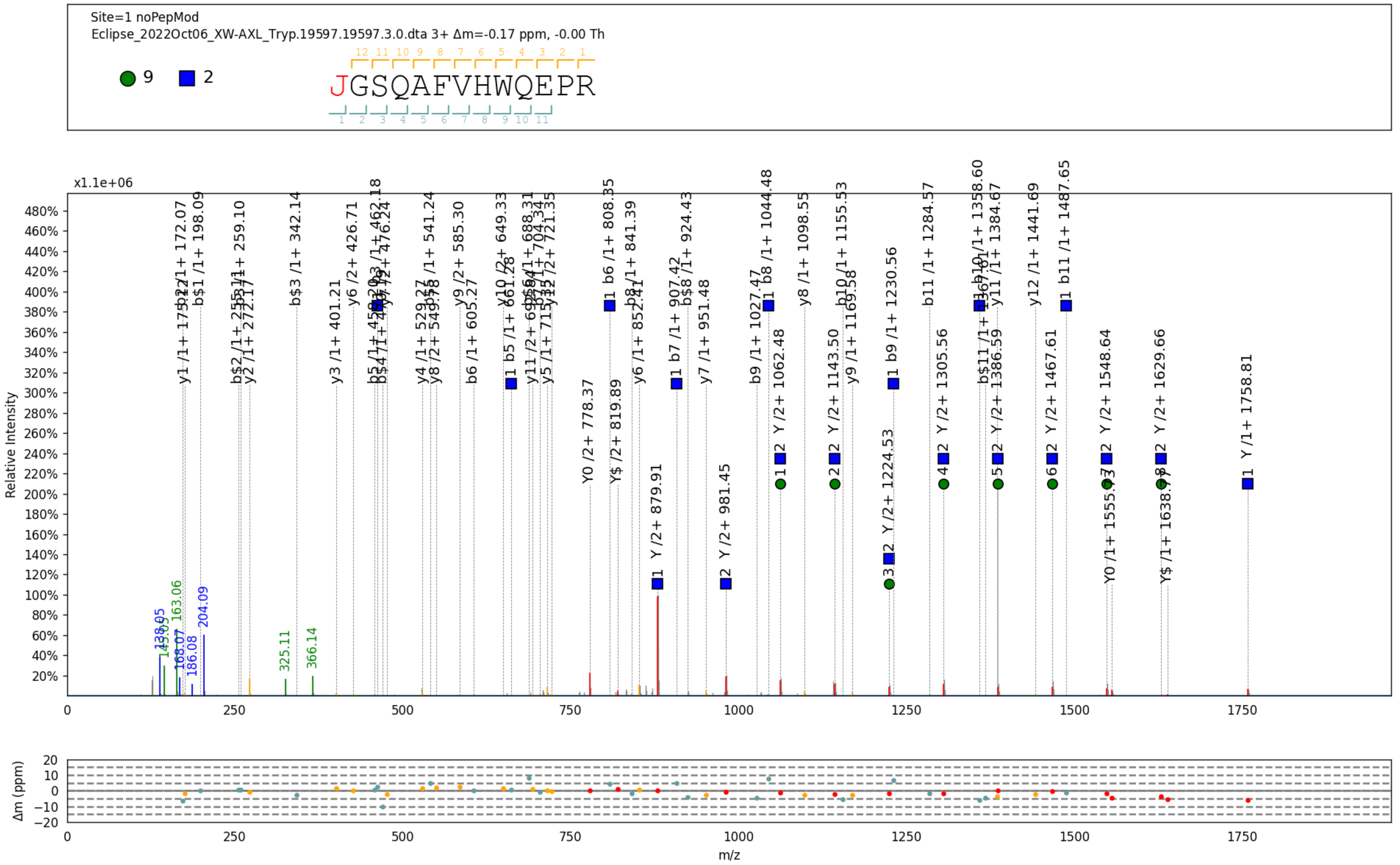
Site=1 noPepMod
Eclipse_2022Oct06_XW-AXL_Tryp.19597.19597.3.0.dta 3+ Δm=-0.17 ppm, -0.00 Th
9
2
JGSQAFVHWQEPR
x1.1e+06
Relative Intensity
Δm (ppm)
m/z

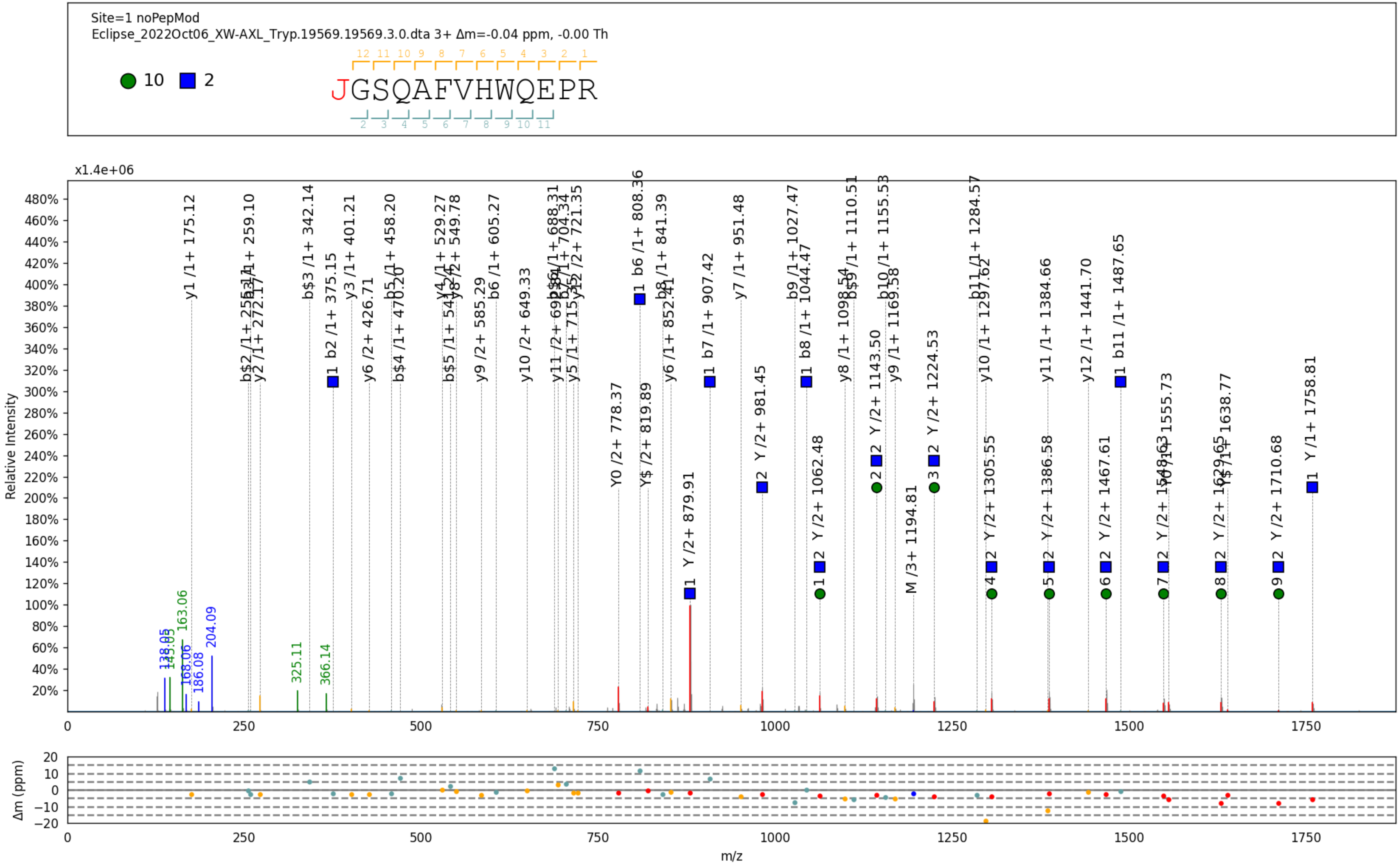

Site=1 noPepMod
Eclipse_2022Oct06_XW-AXL_Tryp.19569.19569.3.0.dta 3+ Δm=-0.04 ppm, -0.00 Th
10
2
JGSQAFVHWQEPR
x1.4e+06
Relative Intensity
Δm (ppm)
m/z

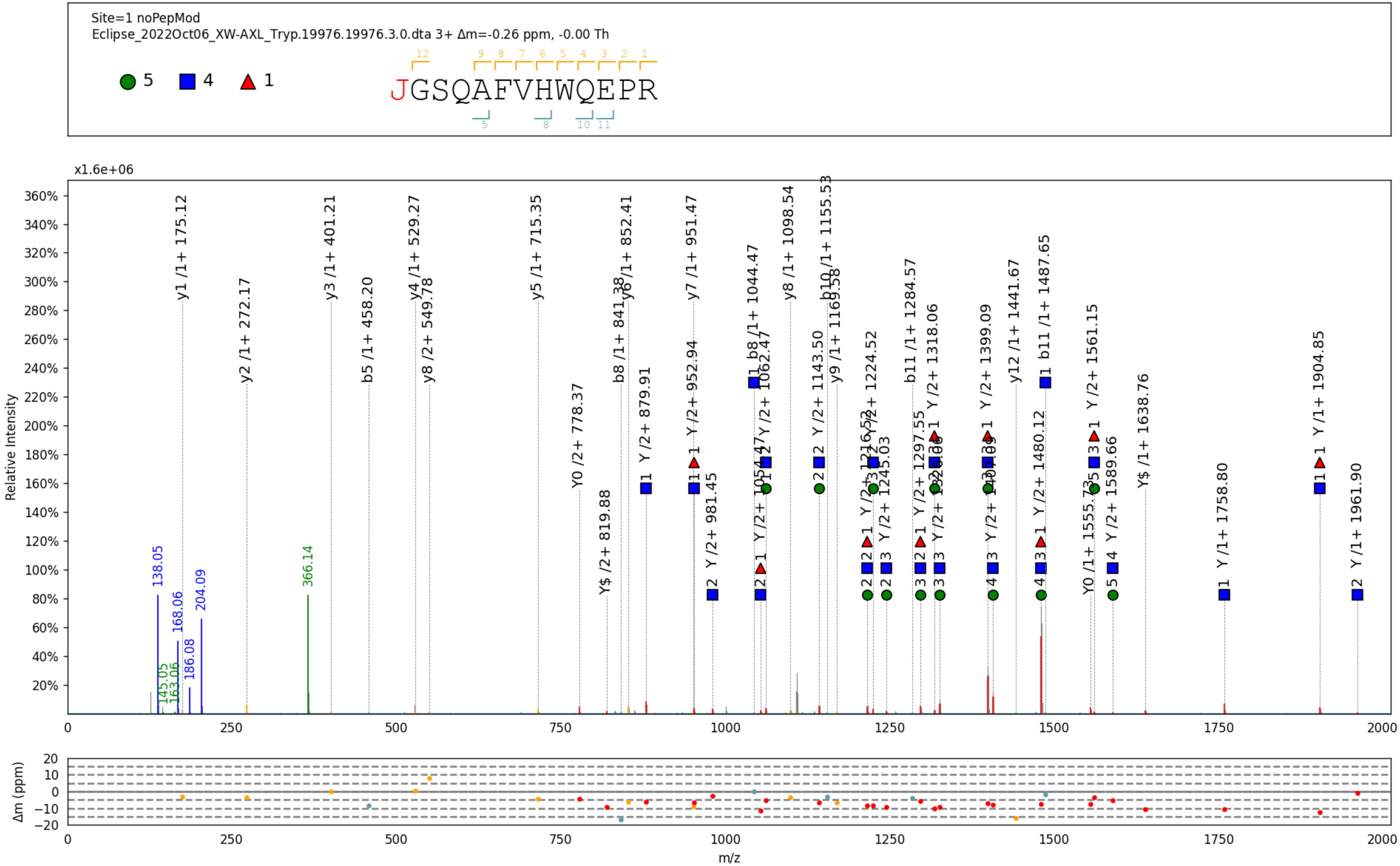
Site=1 noPepMod
Eclipse_2022Oct06_XW-AXL_Tryp.19976.19976.3.0.dta 3+ Δm=-0.26 ppm, -0.00 Th
5
4
1
JGSQAFVHWQEPR
x1.6e+06
Relative Intensity
y1 /1+ 175.12
y2 /1+ 272.17
y3 /1+ 401.21
b5 /1+ 458.20
y4 /1+ 529.27
y8 /2+ 549.78
y5 /1+ 715.35
Y0 /2+ 778.37
Y$ /2+ 819.88
b8 /1+ 841.38
y6 /1+ 852.41
Y /2+ 879.91
y7 /1+ 951.47
Y /2+ 952.94
Y /2+ 981.45
b8 /1+ 1044.47
Y /2+ 1062.47
y8 /1+ 1098.54
Y /2+ 1143.50
b10 /1+ 1155.53
y9 /1+ 1169.58
Y /2+ 1224.52
Y /2+ 1245.03
b11 /1+ 1284.57
Y /2+ 1297.55
Y /2+ 1318.06
Y /2+ 1399.09
y12 /1+ 1441.67
Y /2+ 1480.12
b11 /1+ 1487.65
Y0 /1+ 1555.73
Y /2+ 1561.15
Y /2+ 1589.66
Y$ /1+ 1638.76
Y /1+ 1758.80
Y /1+ 1904.85
Y /1+ 1961.90
138.05
145.05
163.06
168.06
186.08
204.09
366.14
m/z
Δm (ppm)

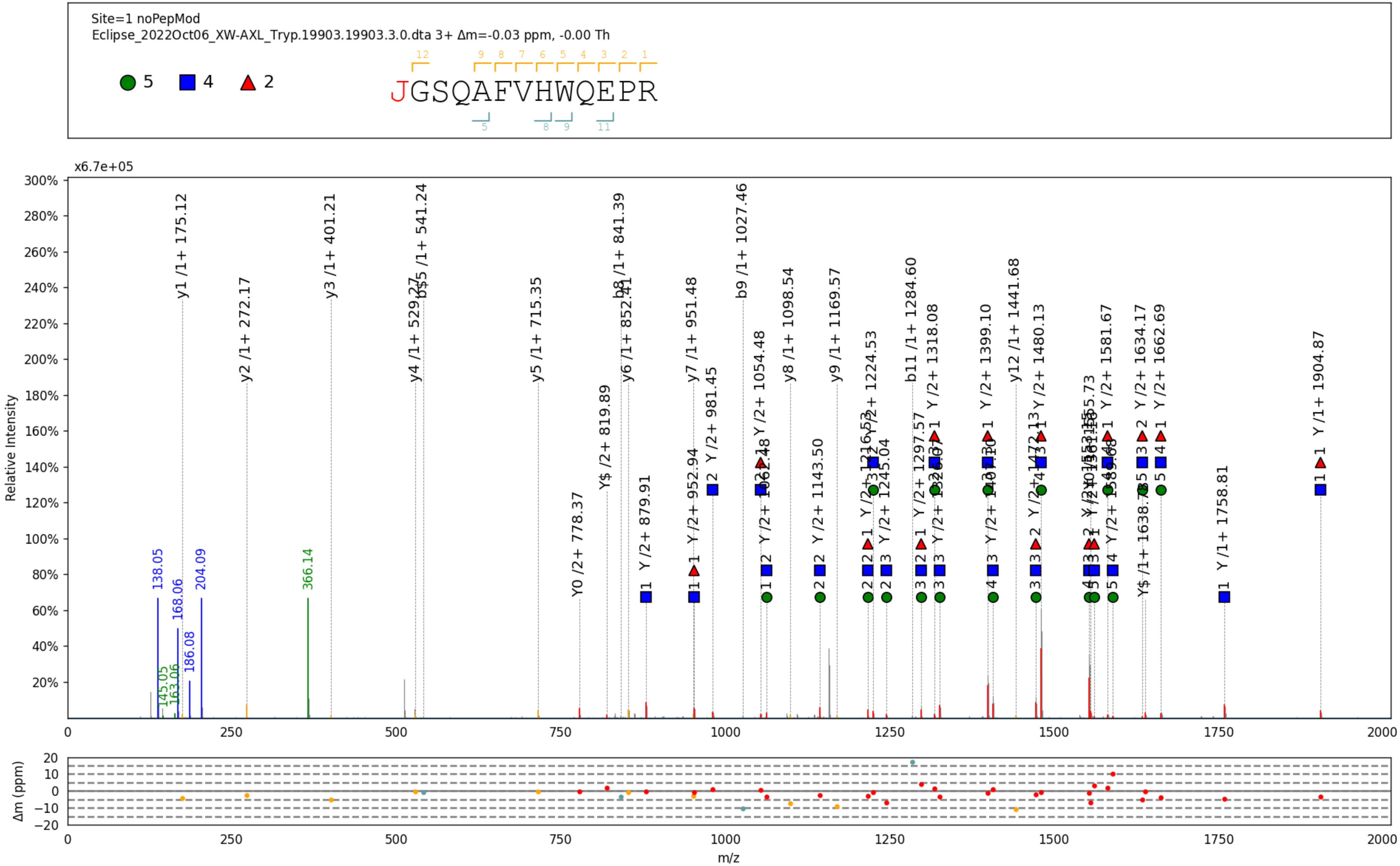

Site=1 noPepMod
Eclipse_2022Oct06_XW-AXL_Tryp.19903.19903.3.0.dta 3+ Δm=-0.03 ppm, -0.00 Th
JGSQAFVHWQEPR
x6.7e+05
Relative Intensity
m/z
Δm (ppm)

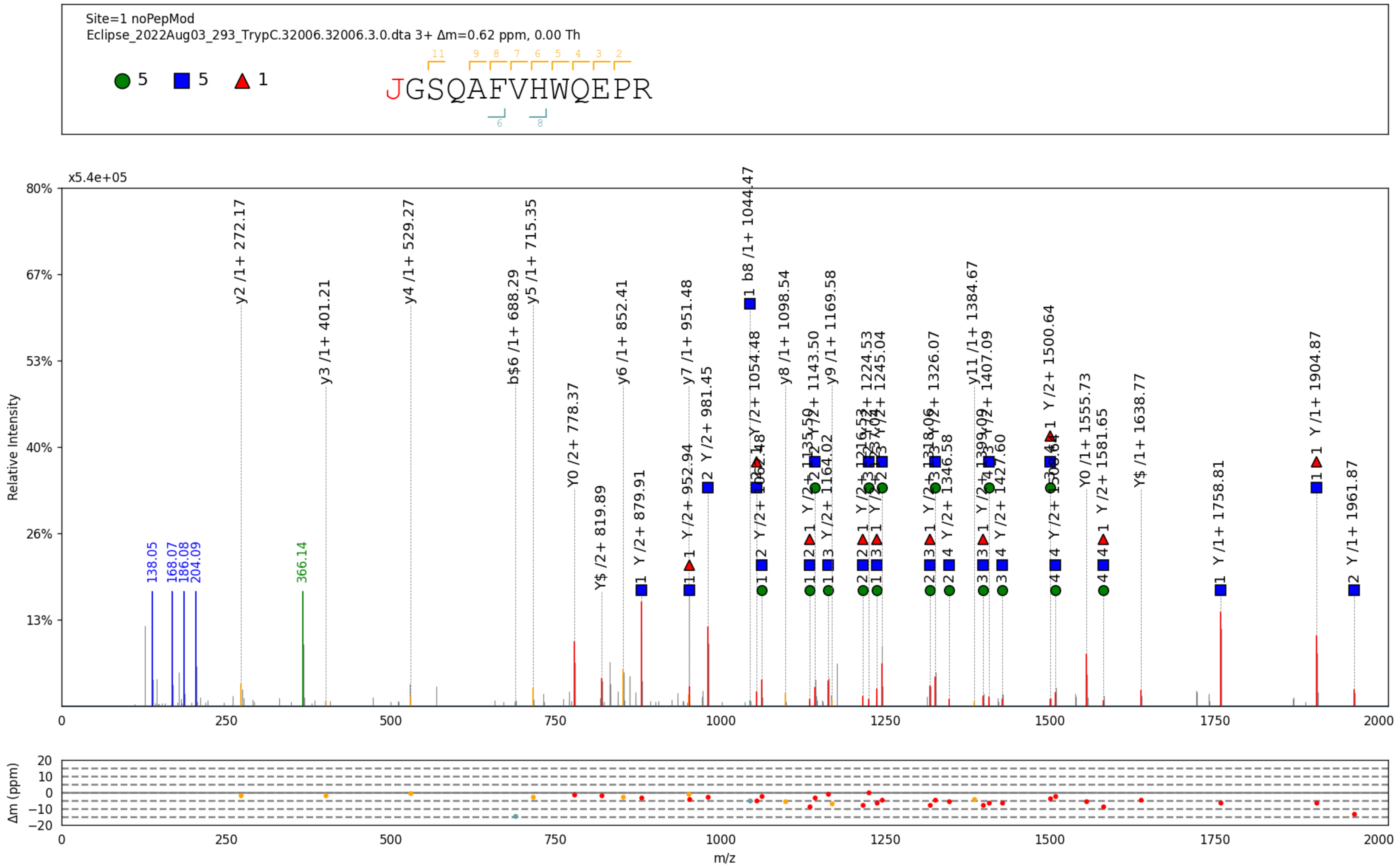

Site=1 noPepMod
Eclipse_2022Aug03_293_TrypC.32006.32006.3.0.dta 3+ Δm=0.62 ppm, 0.00 Th
JGSQAFVHWQEPR
x5.4e+05
Relative Intensity
m/z
Δm (ppm)
y2 /1+ 272.17
y3 /1+ 401.21
y4 /1+ 529.27
b$6 /1+ 688.29
y5 /1+ 715.35
Y0 /2+ 778.37
Y$ /2+ 819.89
y6 /1+ 852.41
Y /2+ 879.91
y7 /1+ 951.48
Y /2+ 952.94
Y /2+ 981.45
b8 /1+ 1044.47
Y /2+ 1054.48
y8 /1+ 1098.54
Y /2+ 1143.50
Y /2+ 1164.02
y9 /1+ 1169.58
Y /2+ 1224.53
Y /2+ 1245.04
Y /2+ 1326.07
Y /2+ 1346.58
y11 /1+ 1384.67
Y /2+ 1407.09
Y /2+ 1427.60
Y /2+ 1500.64
Y0 /1+ 1555.73
Y /2+ 1581.65
Y$ /1+ 1638.77
Y /1+ 1758.81
Y /1+ 1904.87
Y /1+ 1961.87
138.05
168.07
186.08
204.09
366.14

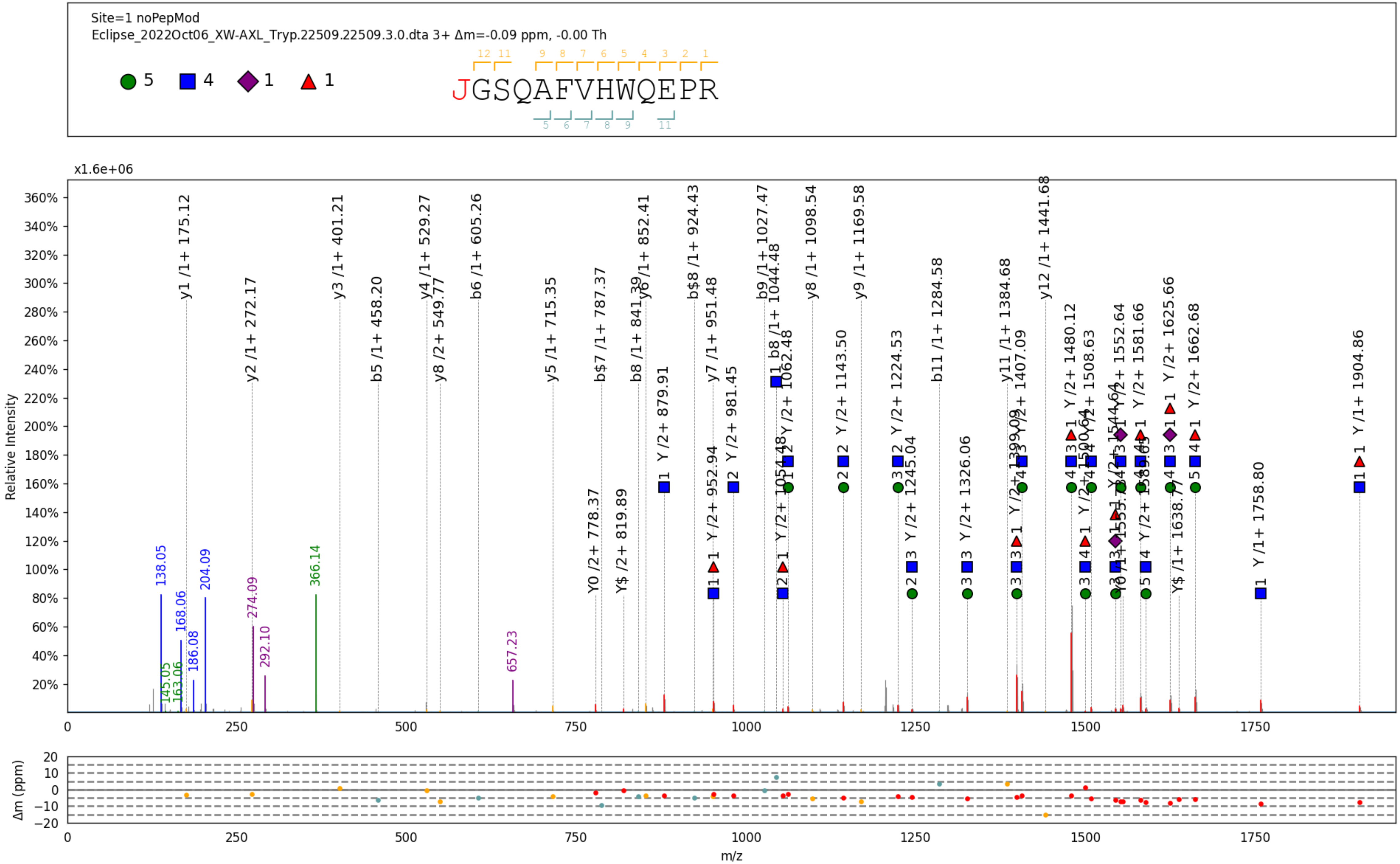

Site=1 noPepMod
Eclipse_2022Oct06_XW-AXL_Tryp.22509.22509.3.0.dta 3+ Δm=-0.09 ppm, -0.00 Th
5 4 1 1
JGSQAFVHWQEPR
x1.6e+06
Relative Intensity
y1 /1+ 175.12
y2 /1+ 272.17
y3 /1+ 401.21
b5 /1+ 458.20
y4 /1+ 529.27
y8 /2+ 549.77
b6 /1+ 605.26
y5 /1+ 715.35
Y0 /2+ 778.37
b$7 /1+ 787.37
Y$ /2+ 819.89
b8 /1+ 841.39
y6 /1+ 852.41
Y /2+ 879.91
b$8 /1+ 924.43
y7 /1+ 951.48
Y /2+ 952.94
Y /2+ 981.45
b9 /1+ 1027.47
b8 /1+ 1044.48
Y /2+ 1054.48
Y /2+ 1062.48
y8 /1+ 1098.54
Y /2+ 1143.50
y9 /1+ 1169.58
Y /2+ 1224.53
Y /2+ 1245.04
b11 /1+ 1284.58
Y /2+ 1326.06
y11 /1+ 1384.68
Y /2+ 1399.09
Y /2+ 1407.09
y12 /1+ 1441.68
Y /2+ 1480.12
Y /2+ 1500.64
Y /2+ 1508.63
Y /2+ 1544.64
Y /2+ 1552.64
Y /2+ 1581.66
Y /2+ 1589.63
Y /2+ 1625.66
Y$ /1+ 1638.77
Y /2+ 1662.68
Y /1+ 1758.80
Y /1+ 1904.86
138.05
145.05
163.06
168.06
186.08
204.09
274.09
292.10
366.14
657.23
Δm (ppm)
m/z

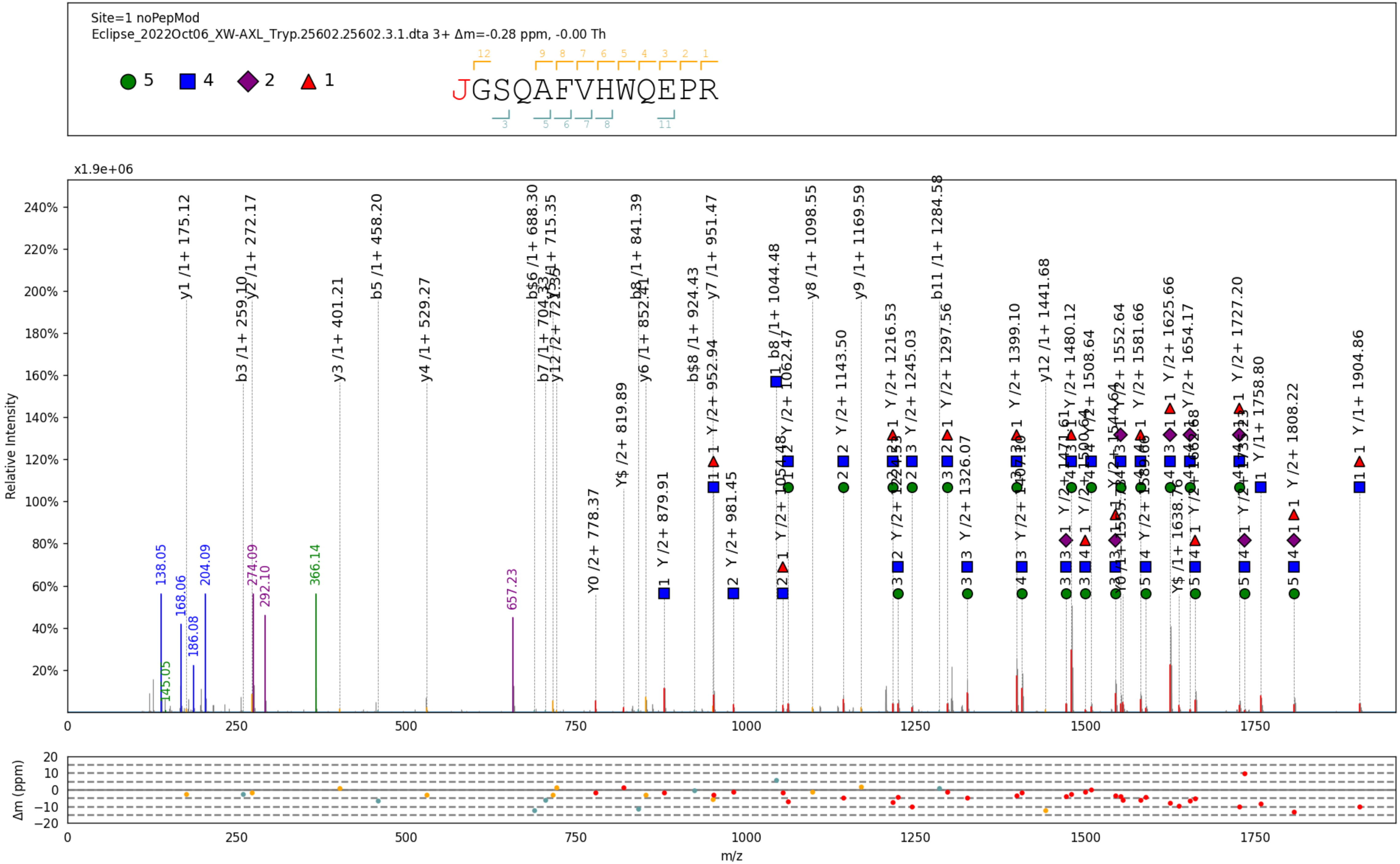

Site=1 noPepMod
Eclipse_2022Oct06_XW-AXL_Tryp.25602.25602.3.1.dta 3+ Δm=-0.28 ppm, -0.00 Th
5
4
2
1
JGSQAFVHWQEPR
x1.9e+06
Relative Intensity
m/z
Δm (ppm)
y1 /1+ 175.12
y2 /1+ 272.17
b3 /1+ 259.10
y3 /1+ 401.21
b5 /1+ 458.20
y4 /1+ 529.27
b$6 /1+ 688.30
b7 /1+ 704.33
y12 /2+ 721.35
Y0 /2+ 778.37
Y$ /2+ 819.89
b8 /1+ 841.39
y6 /1+ 852.41
Y /2+ 879.91
b$8 /1+ 924.43
y7 /1+ 951.47
Y /2+ 952.94
Y /2+ 981.45
b8 /1+ 1044.48
Y /2+ 1062.47
y8 /1+ 1098.55
Y /2+ 1143.50
y9 /1+ 1169.59
Y /2+ 1216.53
Y /2+ 1245.03
b11 /1+ 1284.58
Y /2+ 1297.56
Y /2+ 1326.07
Y /2+ 1399.10
y12 /1+ 1441.68
Y /2+ 1480.12
Y /2+ 1508.64
Y /2+ 1552.64
Y /2+ 1581.66
Y /2+ 1625.66
Y /2+ 1654.17
Y /2+ 1727.20
Y /1+ 1758.80
Y /2+ 1808.22
Y /1+ 1904.86
138.05
168.06
186.08
204.09
145.05
274.09
292.10
366.14
657.23

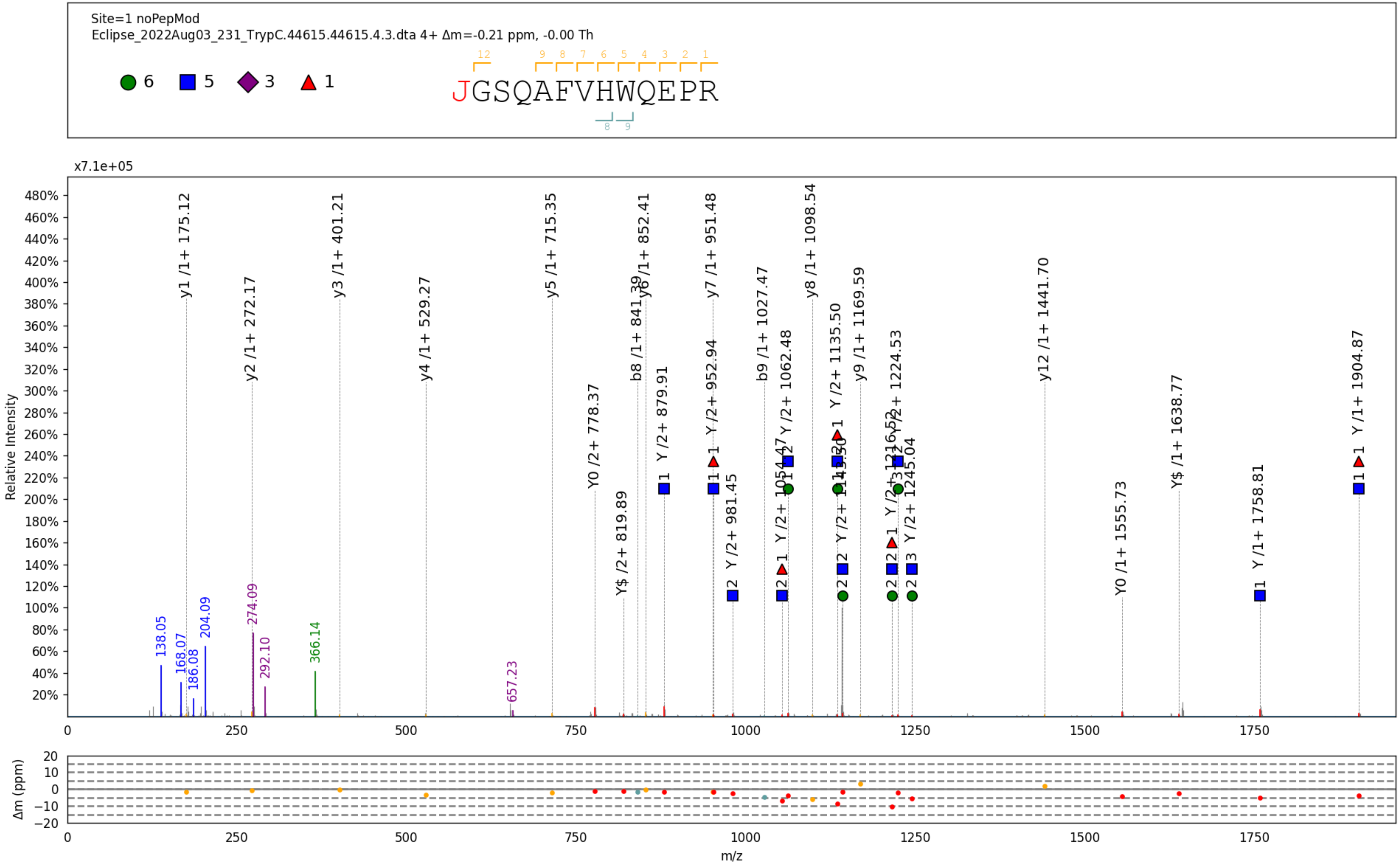

Site=1 noPepMod
Eclipse_2022Aug03_231_TrypC.44615.44615.4.3.dta 4+ Δm=-0.21 ppm, -0.00 Th
6
5
3
1
JGSQAFVHWQEPR
x7.1e+05
Relative Intensity
y1 /1+ 175.12
y2 /1+ 272.17
y3 /1+ 401.21
y4 /1+ 529.27
y5 /1+ 715.35
Y0 /2+ 778.37
Y$ /2+ 819.89
b8 /1+ 841.39
y6 /1+ 852.41
Y /2+ 879.91
y7 /1+ 951.48
Y /2+ 952.94
Y /2+ 981.45
b9 /1+ 1027.47
Y /2+ 1062.48
y8 /1+ 1098.54
Y /2+ 1135.50
y9 /1+ 1169.59
Y /2+ 1224.53
Y /2+ 1245.04
y12 /1+ 1441.70
Y0 /1+ 1555.73
Y$ /1+ 1638.77
Y /1+ 1758.81
Y /1+ 1904.87
138.05
168.07
186.08
204.09
274.09
292.10
366.14
657.23
m/z
Δm (ppm)
